# International Comparison, Risk Assessment, and Prioritisation of 26 Endocrine Disrupting Compounds in Three European River Catchments in the UK, Ireland, and Spain

**DOI:** 10.3390/molecules28165994

**Published:** 2023-08-10

**Authors:** Helena Rapp-Wright, Sara Rodríguez-Mozaz, Diana Álvarez-Muñoz, Damià Barceló, Fiona Regan, Leon P. Barron, Blánaid White

**Affiliations:** 1 DCU Water Institute, Water Hub SG57, Dublin City University, Glasnevin, Dublin 9, Dublin, Ireland; fiona.regan@dcu.ie (F.R.); blanaid.white@dcu.ie (B.W.); 2 School of Chemical Sciences, Dublin City University, Glasnevin, Dublin 9, Dublin, Ireland; 3 MRC Centre for Environment and Health, Environmental Research Group, School of Public Health, Imperial College London, Wood Lane, London W12 0BZ, UK; 4 Catalan Institute for Water Research (ICRA-CERCA), C/Emili Grahit 101, 17003 Girona, Spain; srodriguez@icra.cat (S.R.-M.); dalvarez@icra.cat (D.Á.-M.); dbcqam@cid.csic.es (D.B.); 5 University of Girona (UdG), 17004 Girona, Spain; 6 Institute of Environmental Assessment and Water Research (IDAEA-CSIC), 08034 Barcelona, Spain

**Keywords:** contaminants of emerging concern, water pollution, plasticizers, flame retardants, steroids, LC-MS/MS

## Abstract

Endocrine-disrupting compounds (EDCs) constitute a wide variety of chemistries with diverse properties that may/can pose risks to both humans and the environment. Herein, a total of 26 compounds, including steroids, flame retardants, and plasticizers, were monitored in three major and heavily urbanized river catchments: the R. Liffey (Ireland), the R. Thames (UK), and the R. Ter (Spain), by using a single solid-phase extraction liquid chromatography-mass spectrometry (SPE-LC-MS/MS) method. Occurrence and frequency rates were investigated across all locations over a 10-week period, with the highest concentration obtained for the flame retardant tris(2-chloroethyl) phosphate (TCEP) at 4767 ng∙L^−1^ in the R. Thames in Central London. Geographical variations were observed between sites and were partially explained using principal component analysis (PCA) and hierarchical cluster analysis (HCA). In particular, discrimination between the R. Ter and the R. Thames was observed based on the presence and concentration of flame retardants, benzotriazole, and steroids. Environmental risk assessment (ERA) across sites showed that caffeine, a chemical marker, and bisphenol A (BPA), a plasticizer, were classified as high-risk for the R. Liffey and R. Thames, based on relative risk quotients (rRQs), and that caffeine was classified as high-risk for the R. Ter, based on RQs. The total risks at each location, namely ΣRQ_river,_ and ΣrRQ_river_, were: 361, 455, and 723 for the rivers Liffey, Thames, and Ter, respectively. Caffeine, as expected, was ubiquitous in all 3 urban areas, though with the highest RQ observed in the R. Ter. High contributions of BPA were also observed across the three matrices. Therefore, these two compounds should be prioritized independently of location. This study represents a comprehensive EDC monitoring comparison between different European cities based on a single analytical method, which allowed for a geographically independent ERA prioritization to be performed.

## 1. Introduction

Special attention has been given to endocrine disrupting compounds (EDCs) in recent decades due to their observed adverse effects on organisms or their progeny [1]. These compounds can interfere with the endocrine and hormone systems by disrupting the body’s normal functions, even at very low concentrations (ng∙L^−1^ range [2]). Physicochemically and structurally, EDCs are varied and derive from both natural (e.g., steroid hormones) and synthetic (e.g., plastics, pesticides, etc.) sources. They are widely used in industry (e.g., plasticizers) and domestic activities (e.g., personal care products (PCPs), detergents, surfactants, etc.) and are therefore consumed in large quantities [3]. Since the 1990s, concerns have been raised [4] about environmental contaminants known to have or potentially have endocrine-disrupting properties (EDs). By design, some compounds have long half-lives in the environment, meaning that they do not deteriorate or do so slowly [5,6]. Even compounds not classified as persistent are used and discharged to the environment so frequently that this can give rise to ‘pseudo’ persistence [5,7].

EDC occurrence has been confirmed in different water ecosystems, including surface waters, wastewaters, natural waters, oceans, and even at trace levels in drinking waters [3,8,9], in the ng∙L^−1^–µg∙L^−1^ range [10,11]. Their release into the environment is attributed to different sources, including industrial manufacturing, the human use of materials such as plastics and pesticides, and incomplete removal during treatment in not only wastewater treatment plants (WWTPs) but also drinking water treatment plants (DWTPs) [5]. Their presence is usually higher in rivers with industrial activity, typically as a result of industrial effluent wastewater discharge and/or as a result of these rivers flowing through highly densely populated areas [3], with concentrations reaching high µg∙L^−1^ concentrations. They have also been detected in urban rainfall runoff with almost equivalent concentrations to WWTP effluents [12]. Nevertheless, their sources are constantly changing, as some compounds have been banned for several years and others only recently, resulting in different occurrences and frequencies across countries [7]. However, long-term trends of these compounds depend not only on regulations but also on use, disposal, and production, requiring monitoring over decades, resulting in a lack of studies [13]. Harmful contaminants could leach from landfills and legacy waste and potentially accumulate, impacting ecosystems as well as causing possible synergetic pollution due to their persistence [14,15,16]. Therefore, concentrations vary depending on different factors, including geographical location (e.g., proximity to a WWTP or other pollution sources such as landfill waste), treatment performed in the WWTP, weather conditions (e.g., rainfall or temperature), and seasonal variations (e.g., consumption patterns of the population) [9,14,17]. Consequently, monitoring of these compounds is necessary to evaluate their fate and potential risk.

Predicting whether compounds possess ED properties is challenging given the diversity of compounds. Various effects have been observed, including a reduction in fertility, reproductive organ anomalies, and changes in sexual behaviour in aquatic organisms such as fish and frogs [8]. Negative effects have also been observed in humans, with EDCs shown to be related to the increase of particular metabolic disorders (e.g., obesity, Type 2 diabetes, and cardiovascular disease) and cancer [3,18,19]. Some of these compounds are persistent due to their physicochemical properties (e.g., log K_ow_) and can bioaccumulate [3,20], such as bisphenol A (BPA) in microalgae. Indeed, the potential transfer of this contaminant into other organisms, such as clams entering the food chain, is identified as a potential risk to humans [20]. Consequently, EDCs can accumulate in tissues, causing cumulative, additive, and/or synergic effects [21]. They may not be metabolized, but where they are, transformation products may be equally or even more toxic and persistent than the parent compounds [22]. These effects have been highlighted by the scientific community. Several regulatory bodies across the world have implemented/proposed different strategies for their identification and/or monitoring, such as the Cosmetic Regulation [23] and Plant Protection Products Regulation (PPPR) [24] in the European Union (EU). However, there is no global agreement on their regulation. Existing legislation has been considered insufficient due to the lack of scientific data and knowledge gaps, mainly due to the large chemical diversity [5]. Furthermore, EDCs have the potential to be toxic at extremely low concentrations [1,25,26], which becomes a challenge for their investigation, and accurate thresholds for detecting these analytes have therefore not been established. In the EU, an ED assessment evaluation system has been introduced under Registration, Evaluation, Authorisation, and Restriction of Chemicals (REACH) by the European Chemicals Agency (ECHA) in order to minimize their overall exposure and aim to replace them with safer chemicals (https://echa.europa.eu/ed-assessment, accessed on 4 August 2023). Of a total of 553 compounds on the candidate list that have literature evidence on ED, 60 are classified as Category 1 with evidence of ED activity; 55 as Category 2 with at least some in vitro evidence; and 233 as Category 3, with no scientific basis for inclusion or no data available. There are an additional 205 compounds in a sub-group in Category 3; however, they do not have either the high production volume or the available persistence data required for the assessment [27]. Building on this, some compounds are listed on the “Watch List” (WL) to provide monitoring data to support future prioritization from the EU-Water Framework Directive (WFD), such as oestrogen hormones, for their monitoring of surface waters [28]. The WFD requires EU Member States to achieve good ecological status by establishing river basin management plans (RBMPs) to report parameters such as water quality. However, data obtained from the second RBMP stated that ~60% of surface waters in the EU failed good ecological status and 46% failed chemical status, with 16% considered unknown and not meeting the WFD requirements [29,30]. Developed countries such as the Republic of Ireland reported that almost 50% of their rivers do not present a good status (1589 river water bodies) [31]. Quantitative analytical methods for EDCs in complex environmental matrices usually focus on a limited number of EDCs, typically fewer than 15 in total [17,32,33,34]. In addition, studies generally have paid disproportionately more attention to pharmaceutically related EDC compounds, leaving several others without sufficient knowledge of their environmental and health risks. There is limited data in rivers concerning certain contaminants of emerging concern (CECs), including flame retardants [35], and often, the effects of exposure to environmental mixtures are also highlighted [36], as is the need to perform risk assessments using measure environmental concentrations (MECs) [35]. Monitoring of a larger number of analytes is possible, but it has been performed less frequently in general. This may be due to challenges in achieving broadly comparable and/or suitable sensitivity for all compounds and methods, which in some cases require high sample volumes of up to 1 L, making large-scale continuous monitoring campaigns practically more challenging [17,37,38].

In this study, the hypothesis tested was that currently unmonitored EDCs can be detected at high frequencies and at concentrations that can potentially pose environmental risks in different locations in separate EU states. The objectives were (i) to select three different locations for an international comparison of EDC river contamination based on population size, geographical location, and industrial/agricultural activity; (ii) to perform quantitative analysis of 60 samples (duplicate samples taken weekly over a 10-week period per site) using a single solid-phase extraction liquid chromatography-mass spectrometry (SPE, LC-MS/MS) method for 26 EDCs; (iii) to investigate geographical variation in EDC river contamination; and (iv) to perform an environmental risk assessment to generate a prioritized list of EDCs in aquatic ecosystems. This work represents a comparative international investigation across three countries, including for some EDCs that have been poorly studied to date in selected sites, such as the flame-retardant tris(2-chloroethyl) phosphate (TCEP), for example, and, importantly, explores the prioritization of EDCs from a risk assessment perspective.

## 2. Results and Discussion

### 2.1. Occurrence and Frequency

**Figure 1:**
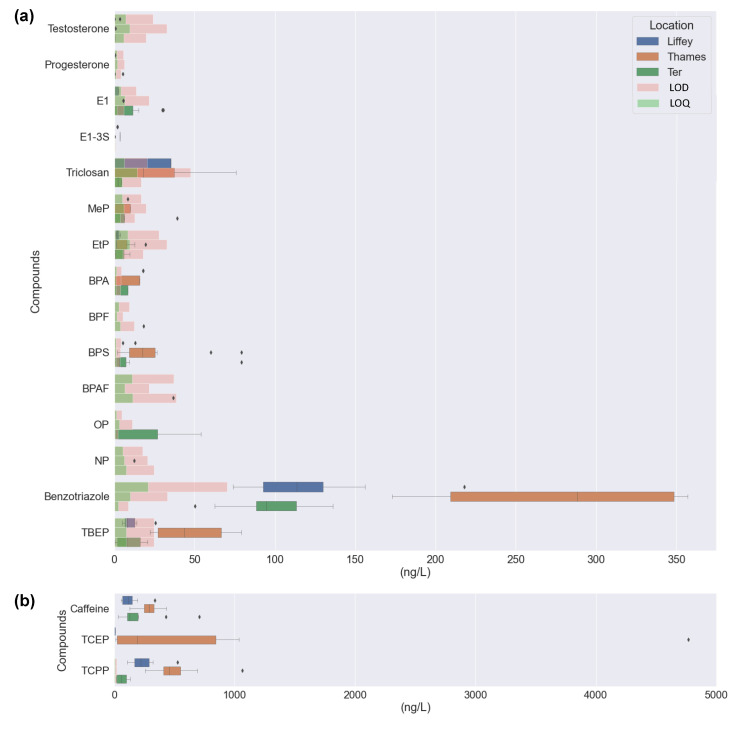
The concentration of selected EDCs in surface waters for all compounds detected (*n* = 10, weeks analysed) for the three areas investigated: Liffey (blue), Thames (orange), and Ter (green), (**a**) for concentrations up to 375 ng·L^−1^ and (**b**) for concentrations detected up to 5000 ng·L^−1^. Boxes represent the interquartile range (IQR), whiskers extend to points that lie within 1.5 IQRs of the lower and upper quartile and dots represent outliers. LODs and LOQs are represented by chart bars in light green and light pink, respectively.

**Figure 2:**
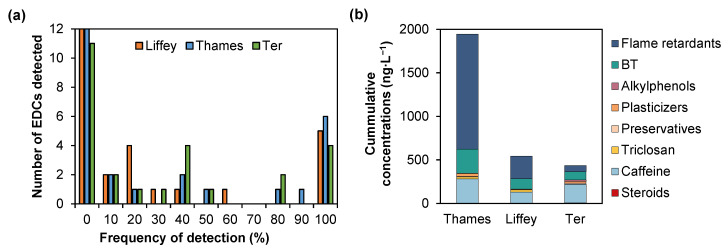
Number of compounds per frequency of detection for the three rivers tested for the sampling campaign (*n* = 10) (**a**) and cumulative concentrations of all EDCs detected for the 10-week sampling campaign for the three rivers investigated. Each colour represents a different class of EDCs detected (**b**).

The occurrence of the EDCs detected in the three locations is presented in Figure 1. Of all 26 compounds analysed, 14 were detected in the R. Liffey and R. Thames, and 15 in the R. Ter. Several compounds were not detected at all across the three sites (Figure 2a). Nevertheless, five (caffeine, EtP, BT, TCPP, and TBEP), six (caffeine, BPS, BT, TCEP, TCPP, and TBEP), and four (caffeine, BT, TCPP, and TBEP) compounds were detected with a 100% frequency for the R. Liffey, Thames, and Ter, respectively. Individual compound frequencies and MECs are available in Tables S6–S8 (Section S2). MECs were determined up to 524 ng∙L^−1^ (TCCP), 4767 ng∙L^−1^ (TCEP), and 705 ng∙L^−1^ (caffeine) for the R. Liffey, Thames, and Ter, respectively. While no riverine flow data were available for both tidal rivers, a dilution factor of ~94% was calculated in the R. Ter (average river flow: 787,465 ± 10,925,99 m^3^∙day^−1^). Overall, of the 26 compounds analysed, only eight, nine, and 10 compounds were quantifiable in all rivers, respectively. While compounds such as PrP, BeP, and BPB were not detected at any location throughout the sampling campaign, compounds such as caffeine and triclosan were detected at all sites at similar frequencies. Overall, the R. Thames presented the highest MECs, mainly from flame retardants, resulting in up to almost 2000 ng∙L^−1^ in total for all compounds (Figure 2b). Lower total MECs were obtained for R. Liffey (543 ng∙L^−1^) and R. Ter (436 ng∙L^−1^). These values followed an expected trend across all areas [39,40], as population density and economic growth have been directly linked with water pollution. This was attributed to the high costs of wastewater treatment and rapid urbanization leading to effluent chemical load increases, which may limit mitigation from receiving water dilution [41].

#### 2.1.1. Steroid Hormones

**Figure 3:**
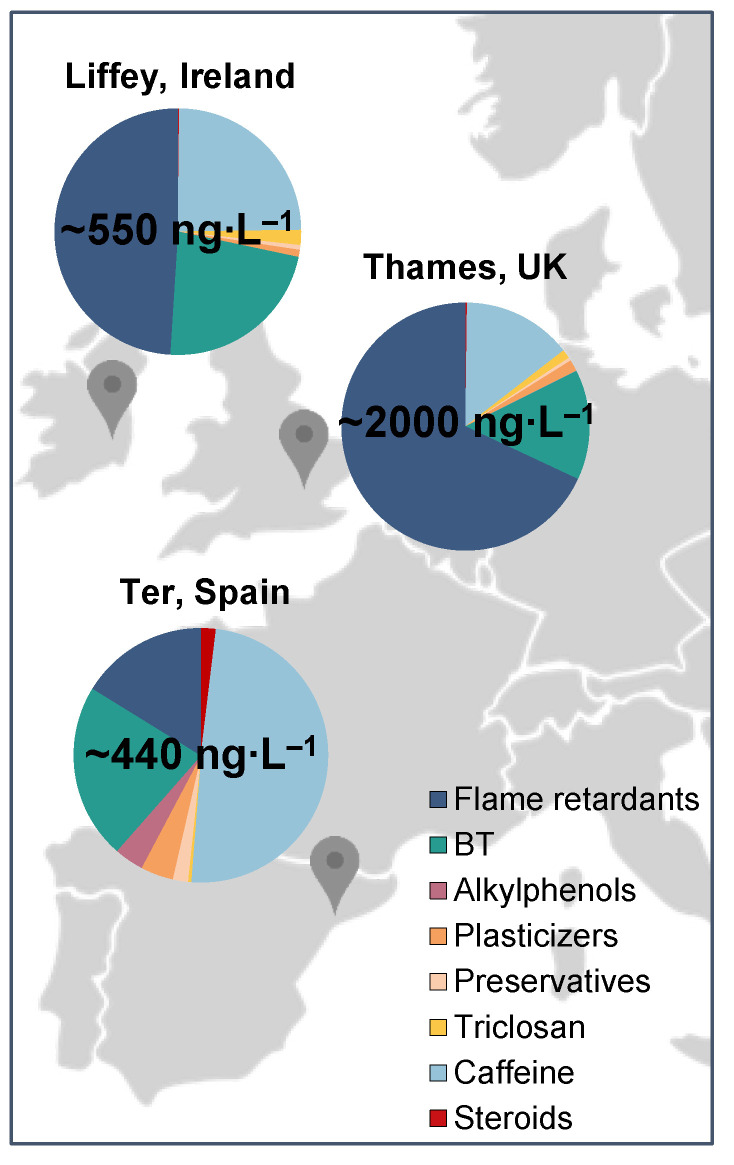
Compound classification of identified EDCs in all three locations: R. Liffey, R. Thames, and R. Ter, showing weekly average cumulative concentration values per location.

Only three hormones (testosterone, progesterone, and E1) were detected at quantifiable concentrations across all eight sites studied. E1-3S was also detected, but <LOQ. This is unsurprising due to the expected low concentrations at which this compound is usually found in the environment (low ng∙L^−1^ range) [42]. However, detection limits stipulated for water in the WFD have not been achieved in this study (e.g., 0.035 ng∙L^−1^ for EE2). These concentrations are very challenging to achieve, resulting in very few studies reporting quantification [43]. In a recent study in Ireland, E1 and EE2 and E1, E2, and E2 were observed in surface waters in an urban and rural area, respectively, but all concentrations were either <LOD or <LOQ [9], aligning with this study’s results. E1 was the only hormone quantified in this study, with the highest concentration in the R. Ter (31 ng∙L^−1^). This agrees with previous studies in the Llobregat River, also in the Catalonia region, which had oestrogens at <LOD [44] and up to 5.81 ng∙L^−1^ [45]. This is likely due to its proximity to the WWTP outlet and the fact that concentrations of 45% remain in effluents using conventional treatments [46,47]. The EU has prohibited hormone use for animal growth since 1981, not only in the member states but also in imports from third countries (Directive 81/602/EEC); however, E1 is also a natural oestrogen. Higher oestrogen concentrations have been found in water bodies and soil from livestock manure [48,49,50]. Compared to the other two more urbanised sites, London and Dublin, the Girona catchment includes some livestock industries. In November 2021, Girona had ~21% and ~12% of the cattle and swine industries, respectively, from the total Catalonia-wide industry [51], with the latter being a high E1 producer during pregnancy [52]. A previous study estimated a higher excretion of E1 from cattle and pigs than from humans per year [52]. On the other hand, no steroids were quantified in the R. Thames samples (E1-3S was only detected at <LOQ as in Figure S6). A previous study reported hormone concentrations between <LOD and up to 17.47 µg∙kg^−1^ in sediment cores in this river [53], and the Environment Agency has predicted high risks in the Thames region associated with predicted E2 values in water of 10–17 ng∙L^−1^ [54]. E1-3S was also detected in the R. Liffey (<3.9 ng∙L^−1^). Only a limited number of studies include the analysis of conjugate steroids in aquatic matrices. However, this compound has been previously detected at concentrations between 12 and 170 ng∙L^−1^ in influent and 7.5 and 34 ng∙L^−1^ in effluent [47], suggesting significant removal during treatment. Sulphate steroid conjugates have low logP and high aqueous solubility values (logS), as seen in Table S1, indicating hydrophilic characteristics leading to their occurrence in water samples. Nevertheless, low concentrations, <10.4 pg∙L^−1^ [55], <LOD [56], and up to 1.46 ng∙L^−1^ in Spain [45], have been reported in surface waters upon dilution once entering the natural environment and are therefore much lower than those determined here. Total contributions of concentration for steroids were 0.2, 0.2, and 2% for the R. Liffey, Thames, and Ter, respectively (Figure 3).

#### 2.1.2. Preservatives

Parabens are widely used as preservatives in various products (e.g., cosmetics, pharmaceuticals, food, etc.) [57]. In this study, out of the four compounds investigated, only MeP and EtP were detected across all locations, while no samples had PrP or BeP detected. MeP is one of the most frequently used parabens in the world [58,59,60] due to its common use in cosmetics [61]. Consistent with this, MeP was present at the highest concentration of all compounds in this category at 39 ng∙L^−1^ in the R. Ter and with a 40% detection frequency (detected between weeks 4 and 7, mid-November to early December). Previously reported concentrations in rivers in the Santiago de Compostela area (Spain) were up to 17 ng∙L^−1^ [62], up to 27 ng∙L^−1^ in the Ebro basin (Spain) [59], and up to 14 ng∙L^−1^ in the Boli River (Taiwan) [60]; all lower than the concentration detected in the R. Thames. Consistent with the MeP findings, the maximum concentration detected of EtP, 20 ng∙L^−1^, was found in the R. Thames as well. It was hypothesized again that this may be because this river serves the highest population of all three locations. In contrast, the highest concentrations of EtP were 4 and 10 ng∙L^−1^ for R. Liffey and R. Ter, respectively. Moreover, no study has reported parabens in these three locations previously. Higher occurrence frequency was obtained compared to MeP, i.e., 100, 90, and 80% for the R. Liffey, R. Thames, and R. Ter, respectively (Figure 2a). Lower concentrations of EtP were also obtained in other studies when MeP and EtP were investigated together, with concentrations up to 3 ng∙L^−1^ in the Santiago de Compostela area (Spain) [62], up to 13 ng∙L^−1^ in the Ebro basin (Spain) [59], and not detected in the Boli river (Taiwan) [60], also consistent with this study. In these reported studies, PrP was detected at concentrations up to 69 ng∙L^−1^ (Santiago de Compostela area, Spain) [62], up to 15 ng∙L^−1^ (Ebro basin, Spain) [59], and 9 ng∙L^−1^ (Boli River, Taiwan) [60]. However, PrP removal rates in the WWTP in the Santiago de Compostela area were higher than 99.9%, and concentrations were found to be higher in the river than the effluent, suggesting its presence in untreated wastewater discharges or leaks from the system [62]. On the other hand, BeP was detected <LOQ [62], up to 1.1 ng∙L^−1^ [59], and not detected [60], respectively, in accordance with our study. In summary, total contributions of concentration for the preservative category were 1, 0.4, and 2% for R. Liffey, R. Thames, and R. Ter, respectively (Figure 3), due to the majority of these compounds not being detected in the samples.

#### 2.1.3. Plasticizers

Of the five BP compounds in the plasticizers category that were analysed, four were detected across all locations. Of these compounds, BPA and BPF were detected, but could not be quantified. Previous studies in Spain have reported similarly low concentrations (e.g., <LOD-61 ng∙L^−1^ for BPA [59]), and it was not detected in Poland even with a lower limit of quantification of 5 ng∙L^−1^ [63]. BPA was also not detected in R. Liffey in previous studies [64]. On the other hand, BPS and BPAF were quantifiable, likely due to better method sensitivity. Maximum concentrations for BPS and BPFA were 79 ng∙L^−1^ equally in both R. Ter and R. Thames and 37 ng∙L^−1^ for the R. Ter, respectively. Previously reported concentrations of BPS in rivers ranged from 1.5–8.7 ng∙L^−1^, not detected-42 ng∙L^−1^, not detected-135 ng∙L^−1^, and not detected-7200 ng∙L^−1^ for rivers in Japan, Korea, China, and India, respectively [65]. BPAF has been previously detected at concentrations ranging between 1.5–16.2 ng∙L^−1^ in surface waters in China [66]. Therefore, concentrations within this study are similar to or higher than those reported elsewhere. Higher concentrations in the R. Ter could be due to the raw discharge of wastewater in small rural areas, as previously reported in Spain, where high levels of BPs were determined in the natural environment but not detected in any of the effluent samples [67]. It should also be remembered that BPA analogues are primarily found in suspended particulate matter (SPM) due to their physicochemical properties [68]. An example was BPAF, which has moderate lipophilicity (logP = 3.4, Table S1), increasing concern with respect to possible bioaccumulation in aquatic organisms and high persistence in the environment [69]. This also suggests that higher concentrations could be found in sediments and/or sludge (after treatment in WWTPs) for these compounds. In the three locations investigated, more than half of the sewage sludge production is used in the agriculture sector as a source of fertilizer due to its high content of organic and inorganic nutrients [70,71,72,73,74].

#### 2.1.4. Alkylphenols

Alkylphenols are one of the most important categories of EDCs due to the high risks associated with them for wildlife and humans. NP and OP belong to Category 1 of the priority list and WFD, as mentioned before [8], and some alkylphenols have been suggested for their inclusion in the next WL chemicals from the WFD to be classified as priority substances for their monitoring in surface waters [8,75]. Their wide use is mainly attributed to the manufacture of surfactants and degradation products of alkylphenol ethoxylates (APEOs) used in household detergents, pesticides, etc. [76]. Therefore, these compounds have been strictly monitored in several countries; however, they are still found at high concentrations in river waters [73,77], including the UK rivers and estuaries, with concentrations of up to 30 µg∙L^−1^ [78]. In this study, only NP was detected in one sample in the R. Thames but was not quantifiable. Up to 20% of NP in UK waters has been estimated to come from textile and clothing wash-off [79]. It has been banned in textile or clothing production in the EU since 2004 as well as in the UK, setting a maximum limit of 0.1% by mass, but products containing this compound are still imported from other countries such as China [80]. A previous report quantified NP at 75 ng∙L^−1^ in the R. Liffey [64] and other alkylphenols not studied here, such as 2,4-dimethylphenol and methyl phenol, present at >70% of samples up to 120 ng∙L^−1^ [81]. OP was also only detected in the R. Ter, with concentrations ranging from 27–54 ng∙L^−1^ and a 40% frequency. These concentrations are lower than previously quantified samples collected alongside the Ter in 2001 before it was banned in the EU, ranging from <60–480 ng∙L^−1^, as well as NP with concentrations between 70–280 ng∙L^−1^ [82], and were not detected in this study. Usually, higher concentrations of NP are detected relative to OP as demonstrated in several European rivers [78] and other countries such as China, suggesting that OP is a minor component in APEOs [76]. These results are in line with R. Thames samples, in which NP was detected (<LOQ) and OP was not detected. However, in R. Ter, NP was not detected in any sample, but OP was quantified in four. This could be associated with the WWTP discharge upstream of the collection point and the seasonal sampling period (winter time). Previously reported studies have detected OP at concentrations up to 91 ng∙L^−1^ and 428 ng∙L^−1^ for NP (Hungary), higher than the ones obtained in this study. However, higher concentrations are usually reported in warmer summer months and are associated with higher production and usage in these periods (e.g., pesticide application) and/or lower WWTP removal rates with higher temperatures [78]. Our study was performed between October and January so results cannot be extrapolated to the summer period. Total contributions in this category varied between locations from 0 (R. Ter) to 4% (R. Thames).

#### 2.1.5. Flame Retardants

Flame retardants are widely used in a variety of products, such as building materials, plastics, motor vehicles, furniture, textiles, electronics, etc. [83,84]. Compounds such as TCEP and TCPP are suspected carcinogens, and recently their concern in the scientific community has increased due to their occurrence in the aquatic environment. Their detection in surface waters has been confirmed extensively worldwide (e.g., in Germany, China, the UK, etc.) at concentrations ranging from ng∙L^−1^ to µg∙L^−1^ due to their incomplete removal from industrial and domestic sewage discharges [84]. This includes TCEP’s early detection in the Llobregat area in river samples in 1988 [85]. In this study, three out of four compounds studied were detected (TBBPA was not detected). TCPP and TBEP were quantifiable in all samples. However, TCEP in the R. Ter was not detected (frequency of 0%), and concentrations in the R. Liffey were <LOQ. This category presented the highest contribution of the total EDC concentrations in R. Liffey and R. Thames (Figure 3), at 49 and 68%, respectively. A contribution of 16% was achieved for the R. Ter, due to the lower concentrations of only two compounds detected. High detection frequencies were obtained for TCPP and TBEP (100%) in all locations, similar to previous studies that reported frequencies between 80 and 99% for this type of compound [84]. This could be associated with their continuous release during manufacture and the fact that TCEP has been replaced by TCPP in Europe since the 1990s [86]. In the UK, TCPP has been quantified at concentrations up to 26,050 ng∙L^−1^ in the river Aire downstream of a WWTP discharge point, higher than effluent concentrations in other countries [87].

The maximum concentration obtained for TCEP throughout the study was 4767 ng∙L^−1^ in the R. Thames. To our knowledge, no data exists for this compound in London. This concentration is in accordance with previously reported concentrations in urban surface waters (e.g., 5698 ng∙L^−1^ in Beijing, China) and is related to high city populations [84], as these are widely used. In this case, R. Thames has the highest population of all three locations. TCPP also presented high concentrations in the R. Thames, up to 1065 ng∙L^−1^, similar to those reported for Beijing (1742 ng∙L^−1^). However, in that study, TBEP was reported at concentrations up to 3617 ng∙L^−1^ [84], significantly higher than here (79 ng∙L^−1^ in the R. Thames). Due to these high concentrations detected, the R. Thames presented the highest cumulative average concentration values of all matrices investigated (Figure 2b), with a total concentration of 2596 ng∙L^−1^.

#### 2.1.6. Other Compounds

Triclosan is an antimicrobial/disinfectant whose maximum concentration detected (76 ng∙L^−1^) was quantified in the R. Thames. This analyte is effectively removed by WWTPs; however, variable removal rates have been reported [62,88], and it sorbs heavily into sediment and activated sludge [89,90]. Measured concentrations here were similar to those reported in previous studies, ranging from 59 in Japan [91] to 95 ng∙L^−1^ in South Wales (UK) [17]. Triclosan could not be determined at comparable concentrations in the R. Liffey, but it was detected above the LOD (21 ng∙L^−1^) in all samples, indicating a high frequency of occurrence. Triclosan concentrations were discernibly lower in the R. Ter relative to the R. Thames, with concentrations <LOQ there. Previous studies have related low concentrations of this compound in surface waters to heavy rains due to the dilutions that occur in the natural environment when comparing dry and wet seasons [92]. This could also be relevant to this study, where the sampling campaign was performed during the autumn-winter period (October-January months), the wet season in Spain. Nevertheless, in the Ebro basin (Spain), previous concentrations ranged from not detected to 2 ng∙L^−1^ in 2010 [59], with more similar results to the ones obtained here. It has not been manufactured in and/or imported to the EU since the beginning of 2017, restricting its use to cosmetic products [93,94]. Moreover, since 2010, it has been banned in Europe as an additive in materials in contact with food [95]. In the R. Thames, this compound presented a strong correlation (R^2^ = 0.71) with precipitation data, as seen in Figure S7, quantifiable after days with high precipitation. As this compound has good removals, its presence could potentially be attributed to the CSOs, but other compounds presented very weak correlations (e.g., caffeine is also a good CSO marker). A contribution of 0.4% of the total concentrations was obtained for the R. Ter location, compared to 2 and 1% for the R. Liffey and Thames (Figure 3). Regarding frequency data, the values obtained were similar across all locations. Results are in accordance with previous data for 139 rivers in the USA, where frequency was calculated at 57.6%, but lower than the ones reported for China, a country with the largest production of PCPs in the world in addition to the largest population [91], of 90% [96].

**Figure 4:**
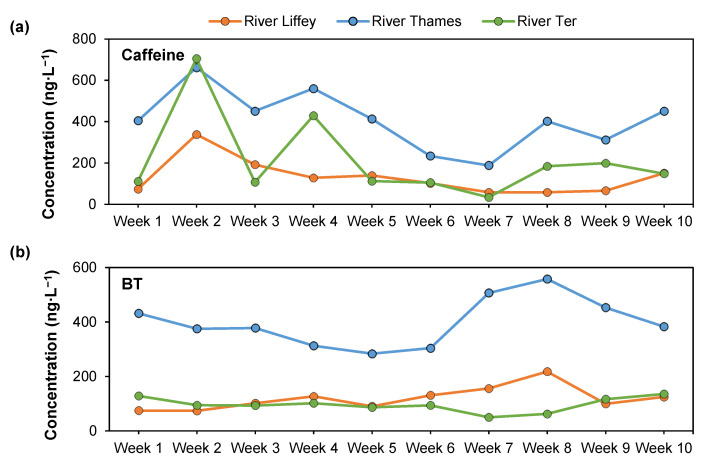
Concentrations (ng∙L^−1^) across all samples analyzed in all three locations: Liffey, Thames, and Ter river waters, for (**a**) caffeine and (**b**) BT. Data from a 10-week period: from the 21st (R. Ter) and 23rd (R. Thames and R. Liffey) of October 2020 to the 20th of January 2021.

The anticorrosive BT was detected with 100% frequency in all three locations, with concentrations ranging between 74–218, 173–357, and 50–136 ng∙L^−1^ for the R. Liffey, R. Thames, and R. Ter, respectively, as observed in Figure 4b. This could be due to its wide use in applications such as household dishwasher detergents, and it is considered the second most frequent contaminant in water due to low removals during treatments in WWTPs [97] and its resistance to biodegradation [98]. It is also related to the tire and rubber industries [82]. Previous concentrations in the R. Liffey were quantified at 309 ng∙L^−1^, higher than those obtained in this study [64]. However, no data has been reported for this compound in the other two rivers, in line with the limited occurrence data available in surface waters when compared to others [99]. The concentrations detected in the R. Ter could be attributed to the close discharge of the WWTP. A previous study measured concentrations of this compound even up to one order of magnitude higher downstream of a WWTP in the R. Leine (Germany) [98]. BT was reported with a 100% frequency, both upstream and downstream, and concentrations ranged between 34–176 and 248–733 ng∙L^−1^, respectively. These concentrations are in line with the ones obtained within this study, where the maximum concentration was 357 ng∙L^−1^ (R. Thames), lower than the maximum reported in Germany [100]. Nevertheless, concentrations in European rivers have been reported up to 6300 ng∙L^−1^ (Switzerland) [101], always with a 100% frequency. This could be attributed to their wide use in Europe, which has a production of 1000–10,000 tons per year [102]. Consequently, even though only one compound has been studied for this category, high contribution percentages to total EDC occurrence were obtained in all rivers, specifically 23, 14, and 22% for the R. Liffey, Thames, and Ter, respectively (Figure 3).

Caffeine was consistently quantifiable in all samples and locations (Figure 2a), with averages of 131 ± 86, 213 ± 203, and 277 ± 93 ng∙L^−1^ for the R. Liffey, Ter, and Thames, respectively. This is unsurprising as it is found in a variety of foods, drugs, and beverages and is the most consumed psychoactive substance in the world, relating the high levels detected to large populations [103,104]. Caffeine has shown some disruptive endocrine activities in fish, suggesting it is a potential xenoestrogen [105,106]. It has a half-life of ~1.5 days in water, but due to its constant discharge, it can act as a persistent chemical, creating dynamic equilibrium [107]. Concentrations in rivers have been reported in Europe up to 880 ng∙L^−1^, but higher concentrations have been detected downstream of WWTPs up to 2400 ng∙L^−1^ [108]. This trend was also observed in this study, as illustrated in Figure 4, where the highest concentrations were shown in R. Ter in one sample in week two of the sampling campaign. Concentrations in the R. Thames were, in most cases, higher than those in the R. Liffey. Caffeine has also been previously reported in the R. Thames at concentrations of 112 ng∙L^−1^ [109] and 389 ng∙L^−1^ in the R. Liffey [64]. However, no concentrations have been previously reported for the R. Ter. High removals of this compound (≥96%) have been reported when using conventional activated sludge as a secondary treatment [109,110], such as in the WWTPs in this study. This suggests a different source for its detection in the river, such as storm runoff and/or CSOs [109,111], and as usual, no caffeine-producing plants are in the area [103]. There are several reasons why this could happen, such as the high use of caffeine by the population, an overflow discharge from the Girona WWTP into the Ter, and the proximity of a major coffee manufacturer ~7 km upstream of the sampling location, which processes annually more than 50,000 tonnes of soluble coffee and ~2400 million coffee capsules annually since 2019. Precipitation data (mm) for the sampling campaign is shown in Figure S5, where Girona (R. Ter) shows the dryer weather overall. The lowest concentration of caffeine obtained is after the highest rainfall recorded, showing the dilution effect of rainfall. This needs a deeper dataset to perform a more insightful statistical analysis on possible correlations using both parameters. 

### 2.2. Identification of Geographical Patterns

**Figure 5:**
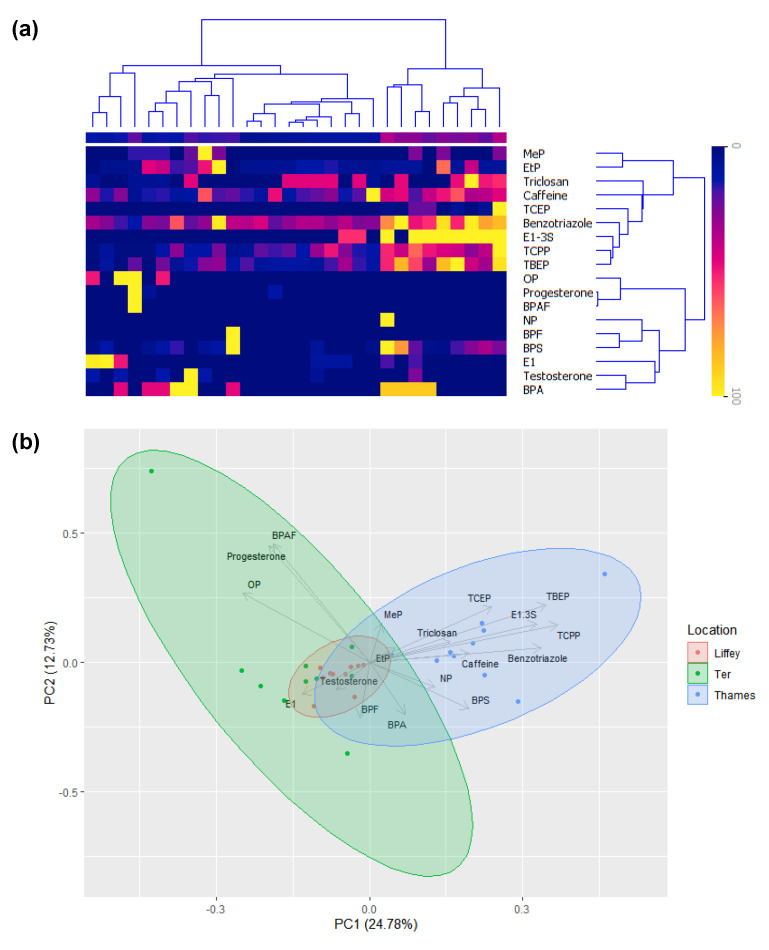
(**a**) Hierarchical cluster analysis (HCA) showing EDC concentrations in the R. Liffey, Thames, and Ter for all 10 weeks sampled (W = week), key (right): darker colors = higher concentrations. (**b**) Principal component analysis (PCA) of the relationship between EDCs detected in the R. Liffey (pink), Ter (green), and Thames (blue), where the percentage explained by the axes is presented in brackets and concentrations were normalized by the compound.

Three cities in Europe were selected for the sampling campaign, and potential geographical variations were explored by hierarchical cluster analysis (HCA). As seen in Figure 5a, three clusters become apparent, grouping the samples mainly by geographical location. Interestingly, only one week of the sampling campaign did not fall within the R. Thames cluster due to low concentrations of compounds such as TCPP and TBEP, and differences for the R. Ter and R. Liffey were mainly due to caffeine and BPA, respectively. Principal component analysis (PCA) was used to understand which compounds explained the variance between the sites (PC1 = 24.8 and PC2 = 12.7%). Results showed three overlapping clusters again (Figure 5b). However, partial separation of the R. Thames cluster was explained by flame retardants (e.g., TBEP, TCEP, and TCPP) and the anticorrosive BT. On the other hand, the main differences in the R. Ter were attributed to hormone steroids (e.g., E1, progesterone, and testosterone). Interestingly, there was no clear differentiation of the R. Liffey from overlapping cluster regions from the other two rivers, and very little variance existed in these samples, probably due to low concentrations in general. These results aligned with the significant differences achieved by ANOVA for the compounds caffeine, BT, and flame retardants, all of which showed higher differences towards the Thames matrix. Nevertheless, the total variance explained only 37.5% (two components), and no complete discrimination patterns were observed, except for the relationship between flame retardants and steroids, shown as well in Figure 3, where the characterization was achieved by percentages of the total contribution based on the concentrations quantified by categories of EDCs. Due to the presence of caffeine at high concentrations, a PCA was performed without this compound; however, only a total of ~39% of the variance was explained, and differences were driven by the same compounds, showing still overlaps between clusters (Figure S8).

### 2.3. Environmental Risk Assessment (ERA)

Half of the compounds from the R. Liffey and R. Ter and a third from the R. Thames were not detected, could not be quantified, or were below 10 ng∙L^−1^, which is the threshold for inclusion in environmental risk assessment guidelines by the European Agency for the Evaluation of Medicinal Products. However, as they possess ED properties, all compounds were considered for assessment regardless of their concentration [112]. Individual RQs are presented in Table 1 for all 18 compounds; as previously mentioned, PNEC_fw_ values were used for R. Thames and R. Liffey, but this is likely to underestimate final RQs, so marine water values were also used for comparison. Based on freshwater values, the highest RQ_fw_ was obtained for caffeine in the three matrices, with 705 as the maximum value obtained overall belonging to the R. Ter. This is due to not only the highest concentrations obtained but also the low PNEC value selected: 1 ng∙L^−1^ was the NOEC concentration in the fish trophic level, according to previous ecotoxicity studies. Reassuringly, most compounds did not pose significant risks, resulting in 64, 57, and 53% of the risk being classified as “insignificant” for the rivers Liffey, Thames, and Ter when using freshwater data. This classification represented nine, eight, and eight compounds, respectively, including compounds such as progesterone, E1-3S, MeP, and BT. Low risks were determined for 7% of the compounds (i.e., one compound) studied in the rivers Liffey and Thames and 27% (i.e., four compounds) for the R. Ter; however, these compounds varied between locations. For example, testosterone, where no discrimination can be made between natural and pharmaceutical occurrences, was classified as low risk for the rivers Thames and Ter and as medium risk in the Liffey due to a higher concentration quantified for this river. Moreover, medium risks were determined as 15, 22, and 13%, respectively, which also varied across the sites. Finally, high risks were associated with a minority of the compounds (14%) for both the Liffey and Thames rivers (i.e., two compounds) and 7% for the R. Ter (i.e., one compound). This is due to the different concentrations quantified across the sites; for example, BPA, which was classified as a high risk for the Liffey and Thames but only a medium risk for the R. Ter. However, caffeine presented high risks at all sites due to its high constant concentrations. When using PNEC_mw_ for the R. Thames and R. Liffey, the high-risk category increased to 36 and 29%, corresponding to five and four compounds, respectively, compared to two compounds when using PNEC_fw_. The additional compounds for R. Liffey were testosterone and triclosan, and for R. Thames were E1, triclosan, and TCEP, increasing all from the medium to high category. The use of marine PNECs will probably overestimate the risk of the site as samples, and it was thought more appropriate to report them as rRQs and use mainly freshwater RQ_fw_.

**Figure 6:**
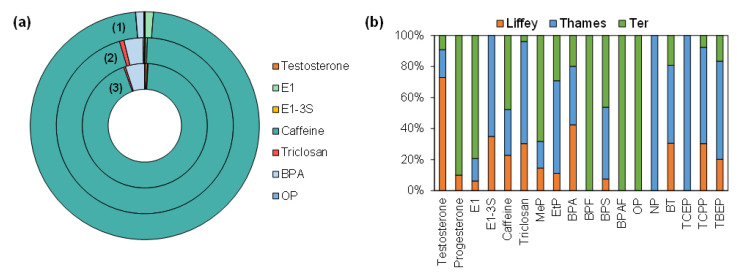
Doughnut plot for the (1) R. Ter, (2) R. Thames, and (3) R. Liffey showing the contribution (%) of individual EDCs detected to the total risk per site (**a**) and stack column plot showing the contribution (%) of individual EDCs detected to the total risk per compound (**b**).

The ERA does not evaluate the combined risk as a result of multiple simultaneous exposures. Consequently, the site risk was calculated, ΣRQ_river_: 361, 455, and 723 for the R. Liffey, Thames, and Ter, respectively using RQs_fw_. All sites posed a very high risk overall, mainly associated with the high concentrations of caffeine in all rivers, contributing to 94, 95, and 97% of the total risk for the rivers Liffey, Thames, and Ter, respectively (Figure 6). Similar risk patterns were observed across all rivers. The second EDC contributing to the highest potential risk, again for all rivers, was BPA, with 5, 3, and 1% for the rivers Liffey, Thames, and Ter, respectively. Lastly, 1% of contributions were obtained for testosterone, triclosan, and E1, respectively. The remaining compounds had extremely low contributions to the total site risk. These results highlight that the following substances: caffeine, BPA, and E1 for the R. Ter; caffeine, BPA, and triclosan for the R. Thames; and caffeine, BPA, and testosterone for the R. Liffey, contributed most to potential environmental risk. These substances should therefore be a primary concern in decision-making regarding the prioritization of chemicals for monitoring, emphasizing caffeine and BPA for all locations, independently of the location investigated. Furthermore, it is worth highlighting the high ΣRQ_river_ value obtained for the R. Ter compared to the other two locations. Even though the Ter presented the least highly populated area of the three rivers, the overall risk was the highest. As mentioned previously, this could be due to the close proximity of the WWTP upstream of the sampling point, again highlighting the importance of different treatment research for the removal of these compounds.

## 3. Materials and Methods

### 3.1. Reagents, Chemicals, and Consumables

LC-MS-optima-grade methanol and water were acquired from Fisher Scientific (Loughborough, UK). Ultrapure water (resistivity of 18.3 MΩ∙cm) was generated from a Millipore Milli-Q water purification system (Millipore, Bedford, MA, USA). Reference standards for estrone (E1), 17-α-ethinyl-estradiol (EE2), estriol (E3), progesterone, testosterone, and tris-(2-chloroisopropyl) phosphate (TCCP) were acquired from LGC Standards Ltd. (Teddington, UK). Bisphenol A (BPA), bisphenol B (BPB), bisphenol S (BPS), bisphenol F (BPF), bisphenol AF (BPAF), triclosan, methylparaben (MeP), benzotriazole (BT), caffeine, tris(2-chloroethyl) phosphate (TCEP), tris(2-butoxyethyl) phosphate (TBEP), estrone-3-sulfate potassium salt (E3-3S), benzyl 4-hydroxybenzoate (BeP), propylparaben (PrP), estriol-3-sulfate (E1-3S), and 3,3′,5,5′-tetrabromobisphenol A (TBBPA) were purchased from Sigma-Aldrich (Steinheim, Germany). Ethylparaben (EtP), 17-β-estradiol (E2), 4-nonylphenol (NP), 4-octylphenol (OP), and 17-α-ethinyl-estradiol (EE2) were obtained from Santa Cruz Biotechnology (Dallas, TX, USA). Compound structures and physicochemical properties selected for monitoring can be found in Table S1 (Section S1). For internal standard reference materials, 17-α-ethinyl-estradiol-d_4_ (EE2-d_4_), 17-β-estradiol-d_2_ (E2-d_2_), estrone-d_4_ (E1-d_4_), methyl 4-hydroxybenzoate-d_4_ (MeP-d_4_), 4-nonylphenol-d_4_ (NP-d_4_), 4-octylphenol-d_17_ (OP-d_17_), benzotriazole-d_4_ (BT-d_4_), bisphenol A-d_4_ (BPA-d_4_) and caffeine-d_3_ were supplied by CDN Isotopes (Qmx Laboratories, Essex, UK). Triphenyl phosphate-d_15_ was obtained from Sigma-Aldrich (Steinheim, Germany), ethyl 4-hydroxybenzoate-ring-^13^C_6_ solution from Fluka (Sigma-Aldrich, Steinheim, Germany), and progesterone-d_9_ from LGC Standards Ltd. (Teddington, UK). All reference standards used had ≥95% purity. Stock standard solutions, stable isotope-labelled internal standards (SIL-IS), and surrogate standard solutions were prepared at a concentration of 1 mg∙L^−1^ in methanol and stored at −20 °C. Further diluted solutions were prepared daily by mixing standards in a mixture of methanol: water (15:85, *v*/*v*) before any sample analysis or method performance experiments.

### 3.2. Site Locations

River water grab samples were collected weekly in three different cities on the European continent (Spain, the UK, and Ireland). The R. Liffey was selected from Dublin (the capital city of the Republic of Ireland), which accounts for 25% of the national population (i.e., 1173,179 in 2016, as per the last census [115,116]). Samples were taken in the city centre at O’Connell Bridge (53°20′49.2″ N; 6°15′39.8″ W). Ringsend WWTP discharges effluent into the lower Liffey Estuary into Dublin Bay, ~4 km downstream of the sampling collection point, as seen in Figure S1. It serves a population equivalent (PE) of 1,640,000 and provides primary and secondary treatment. This portion of the river is brackish and tidal. Although significant dilution of micropollutant concentrations occurs at the WWTP discharge point, upstream tidal flow carries contaminants into the sampling region twice daily. Despite this, water quality was considered “good” downstream and upstream of both the Liffey Estuary and Dublin Bay at the time of the study under the WFD (2020). The WWTP has an average hydraulic loading capacity of 458,641 m^3^∙day^−1^, with 95 incidents recorded in 2020, including uncontrolled releases and spillages [117]. Drinking water for Dublin is abstracted inland from the R. Liffey or Ballymore Eustace.

The R. Thames sampling site at Gabriel’s Pier (51°30′30.3″ N; 0°06′36.7″ W) was located in Central London (the capital city of the UK; population: ~9,176,530 people) [118] and in line with previous studies on pharmaceutical compounds [109]. There are six combined sewer overflow (CSO) vents close by in both directions, as in Figure S2 [119]. Releases from the combination of storm flow with treated and untreated wastewater are very frequent, even with low precipitation in the city. Furthermore, treated effluents from several WWTPs in London (e.g., Beckton, Mogden, Riverside, and Crossness) discharge directly into this river, serving a population of ~91% of Greater London [109]. The site monitored on the R. Thames is also tidal and brackish; conductivity over the sampling period can be seen in Figure S3, and data was used from Environment Agency River monitoring platforms. Drinking water for London is abstracted from the non-tidal reaches above Teddington Lock, to the west of the city, as well as from the R. Lea to the north of the city, and the abstracted water is stored in large reservoirs in both locations.

In Spain, water samples were collected from the R. Ter in Girona, ~1250 m downstream of the Girona WWTP (42°01′41.4″ N; 2°50′53.5″ E) (Figure S4), which serves the entire city of Girona as well as surrounding urban areas with little industrial activity and a total residential population of ~102,666 [120]. This WWTP receives untreated hospital wastewater (1000–1500 m^3^∙day^−1^) and municipal wastewater from the city (45,000–55,000 m^3^∙day^−1^) [121], with a PE of 200,000 inhabitants and an average daily inflow of 35,000 m^3^∙day^−1^. It comprises up to secondary treatment, including activated sludge [122]. This river also provides a source of drinking water, abstracted from a system of three reservoirs (Sau-Susqueda-Pasteral), for the Girona region and the Barcelona metropolitan area and further treated in the Cardedeu DWTP (Barcelona) [82,121]. River flow data for this site was taken from the Catalan Water Agency (Agència Catalana de l’Aigua) platform for the sampling dates and can be seen in Table S2. Unfortunately, no riverine flow data were available for both sampling regions of the R. Thames and R. Liffey, and dilution could not be reliably calculated.

### 3.3. Sample Collection and Preparation

All samples were collected weekly during a 10–week period from October 2020 to January 2021 using 500 mL Nalgene bottles (Thermo Scientific, Rochester, NY, USA) during the morning between 9:00 and 11:00 a.m. and mostly on the same dates (Table S3). Bottles were pre-rinsed twice with methanol, ultrapure water, and river water separately prior to sampling. Grab samples were collected in duplicate and transported to the respective laboratories in a cool box filled with ice packs. On arrival, samples were filtered using a 0.7 µm glass microfiber filter (Whatman^®^, Grade GF/F, Fisher Scientific Ltd., Loughborough, UK), followed by a 0.45 µm polyvinylidene fluoride (PVDF) membrane filter (Millipore; Billerica, MA, USA); details of the filtration are in the Supplementary Materials. After filtering, samples were stored under −20 °C freezing conditions prior to transportation or analysis. Samples collected in Dublin and London were shipped frozen to the Catalan Institute for Water Research (ICRA) at the Science and Technological Park of the University of Girona (Parc Científic I Tecnologic de la Universitat de Girona, Spain) for analysis. Samples were extracted according to Becker et al. (2017) [46]. Briefly, SPE was carried out using a vacuum manifold (Phenomenex, Cheshire, UK) and Strata™-X cartridges (200 mg, 6 mL barrel, 33 µm, Phenomenex, Aliso Viejo, CA, USA). Conditioning was with 5 mL of methanol and 5 mL of ultrapure water at a pH < 2. A sample volume of 100 mL (pre-spiked with surrogate standards at a concentration of 500 ng∙L^−1^ where appropriate; concentration based on 50 ppb in the final extract) was loaded at ~1 mL∙min^−1^. Cartridges were washed using 6 mL of ultrapure water and dried under vacuum for 5 min. Elution was performed with 7 mL of dichloromethane:methanol (50:50, *v*/*v*). Extracts were evaporated to near dryness under N_2_ and reconstituted in a mixture of methanol:water (15:85, *v*/*v*) to 1 mL. Finally, SIL-IS was added to the extract as internal standards at a final concentration of 50 µg∙L^−1^. For quantification purposes, calibration curves were prepared at the following concentrations: 0.5, 1, 5, 10, 25, 50, 100, and 200 µg∙L^−1^ and SIL-IS was added at a final concentration of 50 µg∙L^−1^ in a final volume of 1 mL in methanol:water (15:85, *v*/*v*). Recovery values were used to correct calculations of final analyte concentrations; therefore, no SPE was performed on calibration points. The peak area ratio was used for quantification, and details of the SIL-IS used can be seen in Table S4.

### 3.4. Instrumental Analysis

Analysis was performed according to Becker et al. (2017) [46], and further details are given in Supplementary Materials, Section S1. Briefly, liquid chromatography (LC) separations were performed on a Luna Omega C_18_ analytical column (100 × 2.1 mm, 1.6 µm particle size) from Phenomenex (Torrance, CA, USA). The LC system comprised an Accela 4 Open AS autosampler and a quaternary pump from Thermo Fisher Scientific (San Jose, CA, USA). Mass spectrometry was performed using a Thermo TSQ Vantage triple quadrupole mass spectrometer equipped with an electrospray ionization (ESI) source operating in separate runs in positive or negative ionization mode. The acquisition of the selected compounds was achieved in multiple reaction monitoring (MRM) modes, where two transitions were selected for ion confirmation, with the most abundant transition used for quantification and the other one for qualification/confirmation purposes. MRM transitions can be observed in Table S4 (Section S1) for both negative and positive ESI polarities. Data acquisition and processing were performed using Xcalibur v4.3 software (Thermo Fisher Scientific, San Jose, CA, USA).

### 3.5. Method Performance

Although the analytical method was previously validated for different sample matrices (river, tap water, effluent, and influent wastewater), method detection and quantification limits (LOD and LOQ, respectively) were re-evaluated for matrices from each sample location (i.e., Ireland, Spain, and the UK) as samples derived from both brackish and freshwater rivers (Table S5). Maximum LODs and LOQs were <21 and <70 (triclosan), <12 and <39 (EE2), and <18 and <61 (BPF) ng∙L^−1^, respectively, for the three matrices. Recoveries from samples in all three locations are presented in Table S5 and used to correct calculations of final analyte concentrations due to possibly different matrix effects, with averages of 85 ± 29%, 77 ± 33%, and 89 ± 35%, respectively. Samples for method performance were prepared in triplicate for every water type by spiking the standard solution at 500 ng∙L^−1^ in three different water samples from each matrix.

### 3.6. Environmental Risk Assessment (ERA)

The risk associated with the contaminants detected at all sites was assessed by calculating risk quotients (RQs). For their calculation, the highest concentration quantified for the compound per site was used as the MEC value; if any compound was detected below the LOQ, half of the method LOQ was used as the MEC [9,123]. The MEC value was then divided by the lowest predicted no-effect concentration (PNEC) value (obtained from the NORMAN Ecotoxicology database and literature review (Table 1)). As both the R. Thames and R. Liffey are brackish and tidal rivers, PNEC freshwater values (PNEC_fw_) may underestimate the environmental risk, so RQs are reported as relative RQs (rRQs) using PNEC_fw_ for these two sites. R. Thames conductivity during the sampling times (9:00–11:00 a.m.) was ≤739 µS·cm^−1^ indicating low saline conditions (1000–10,000 µS·cm^−1^) [124,125,126,127,128,129]. A previous study showed the influx/efflux of the tidal cycle on the same sampling location ranging up to 1000 µS·cm^−1^ [109], and conductivity over the sampling period can be seen in Figure S3. No conductivity data was available for the R. Liffey during the sampling campaign, but all samples were taken within 10 cm of the surface, where water remains as a freshwater area at the top layer (~15 cm) as salinity increases with depth [124,126]. Rainfall also decreases salinity levels [130,131], and the top water layer has been shown to have lower salinity in wet seasons [126]. Daily total precipitation (mm) data can be found in Figure S5 for all rivers, where precipitation occurred in close proximity or even on the day of sampling for the R. Liffey and R. Thames. Precipitation data was obtained from open data sources: AEMET OpenData (Agencia Estatal de Metereología) for Girona (Girona Airport Station), the National Oceanic and Atmospheric Administration (NOAA) Integrated Surface Database (ISD) (National Centres for Environmental Information) for London (Heathrow station, code = 037720-99999), and MET Éireann (The Irish Meteorological Service, https://www.met.ie, accessed on 6 February 2023) for Dublin (Ringsend Station).

PNEC data for marine waters (PNEC_mw_) were also used to evaluate the worst-case scenario as PNEC_fw_/10. For RQ level classification, RQs below 0.1 were considered to pose an insignificant risk; 0.1 < RQ < 1.0 were considered to carry a low risk; 1.0 < RQ < 10.0, a medium risk was assigned; and where RQ > 10.0, a high environmental risk was assigned. To assess the potential risk of the entire site, total risks and relative risks were calculated (ΣRQ_site_ and ΣrRQ_site_) for the R. Ter, R. Thames, and R. Liffey respectively. The estimation of the contribution of each compound to the site was also calculated by dividing the RQ of the compound by the total risk of the investigated area.

### 3.7. Statistical and Data Analysis

A statistical analysis was performed to assess any geographical variation. Mean concentration values by categories of contaminants (Table S1) were used where the normality of the data was tested by the Shapiro–Wilk test applying a *p* < 0.05 significance level. Analysis of variance (ANOVA) with the post hoc Tuckey’s test (*p* < 0.05) and independent t-test were used for parametric data where necessary. Kruskal–Wallis ANOVA by ranks and independent-sample Mann–Whitney U tests were used for non-parametric data. Concentrations below the LOQ were assumed to be half of the value of the limit (specific compound and specific matrix), and not-detected compounds were set to zero [132]. Microsoft^®^ Office Excel (WA, USA), IBM^®^ SPSS Statistics v27 (New York, NY, USA), R v4.0.5, RStudio v1.4.1106, Python version 3.7.9, and Orange Visual Programming freeware (Bioinformatics Lab at the University of Ljubljana, Slovenia) were utilized.

## 4. Conclusions

An international study of 26 EDCs was carried out across three rivers in the Republic of Ireland (R. Liffey), the United Kingdom (R. Thames), and Spain (R. Ter) over a 10-week period. A total of 14 compounds were detected for the rivers Liffey and Thames and 15 for the Ter, where concentrations were up to 524 ng∙L^−1^ (TCCP), 4767 ng∙L^−1^ (TCEP), and 705 ng∙L^−1^ (caffeine), respectively. Overall, higher concentrations were measured in the R. Thames, where cumulative weekly average concentration values of up to 2000 ng∙L^−1^ were obtained. This could be attributed to the high-density population area, the central catchment sampling point, and the CSOs next to the sampling location. However, only triclosan presented a strong correlation (R^2^ = 0.71) with precipitation data. Caffeine was obtained at the highest concentration in the R. Ter, even though the city’s population is much lower, probably due to proximity to a WWTP effluent downstream of the collection point. Some geographical variations across sites generally separated well when HCA and PCA were applied and were explained by four categories: plasticizers, caffeine, flame retardants, and BT. An environmental risk assessment was performed, with high risks associated with two compounds for the R. Liffey and Thames and one for the R. Ter. The highest RQ was calculated for caffeine In the R. Ter (RQ = 705) and generally explained most of the combined RQs in all samples across sites, along with BPA. Consequently, these compounds should be prioritized to define future policy development to protect and enhance water quality across different geographical locations. A substance priority was determined by location, with the following EDCs identified: (a) caffeine, BPA, and E1 for the R. Ter; (b) caffeine, BPA, and triclosan for the R. Thames; and (c) caffeine, BPA, and testosterone for the R. Liffey.

## Figures and Tables

**Table 1:** MEC and PNEC selected values for environmental risk quotients (RQ) calculation and level classification of EDCs per site location, where insignificant (I), low (L), medium (M), or high (H), and total risk per site (ΣRQ_site_) for freshwaters (fw) and marine waters (mw) where required.

Compounds	PNEC_fw_ (ng·L^−1^)	PNEC_mw_ (ng·L^−1^)	Liffey					Thames					Ter		
			MEC (ng·L^−1^)	rRQ_fw_	Risk_fw_	rRQ_mw_	Risk_mw_	MEC (ng·L^−1^)	rRQ_fw_	Risk_fw_	rRQ_mw_	Risk_mw_	MEC (ng·L^−1^)	RQ_fw_	Risk_fw_
Testosterone	1.5 [113]	0.15	3.63	2.42	M	24.2	H	0.900	0.6	L	6.00	M	0.459 ^a^	0.306	L
Progesterone	1000 ^b^	100	0.600	6 × 10^−4^	I	6 × 10^−3^	I	-	-	-	-	-	5.40	5 × 10^−3^	I
E1	3.6 ^b^	0.36	2.46 ^a^	0.683	L	6.83	M	5.60 ^a^	1.56	M	15.6	H	30.6	8.50	M
E1-3S	20,500 ^b^	2050	1.95 ^a^	1 × 10^−4^	I	1 × 10^−3^	I	3.62 ^a^	2 × 10^−4^	I	2 × 10^−3^	I	-	-	-
Caffeine	1 [113]	0.1	338	338	H	3380	H	432	432	H	4320	H	705	705	H
Triclosan	20 ^b^	2	35.1 ^a^	1.75	M	17.5	H	75.8	3.79	M	37.9	H	4.38 ^a^	0.219	L
MeP	5000 ^b^	500	8.41 ^a^	2 × 10^−3^	I	2 × 10^−2^	I	9.88 ^a^	2 × 10^−3^	I	2 × 10^−2^	I	39.2	8 × 10^−3^	I
EtP	2500 [58]	250	3.69	1 × 10^−3^	I	1 × 10^−2^	I	19.5	8 × 10^−3^	I	8 × 10^−2^	I	9.53	4 × 10^−3^	I
BPA	1 [114]	0.1	18.0 ^a^	18.0	H	180	H	15.9 ^a^	15.9	H	159	H	8.41 ^a^	8.41	M
BPF	840 [68]	84	-	-	-	-	-	-	-	-	-	-	18.3 ^a^	2 × 10^−2^	I
BPS	6900 [66]	690	13.0	2 × 10^−3^	I	2 × 10^−2^	I	79.3	1 × 10^−2^	I	0.11	L	79.3	1 × 10^−2^	I
BPAF	230 [66,68]	23	-	-	-	-	-	-	-	-	-	-	36.9	0.160	L
OP	100 ^b^	10	-	-	-	-	-	-	-	-	-	-	54.1	0.541	L
NP	300 ^b^	30	-	-	-	-	-	12.3 ^a^	4 × 10^−2^	I	0.41	L	-	-	-
BT	7770 ^b^	777	218	2.8 × 10^−2^	I	0.3	L	357	5 × 10^−2^	I	0.46	L	136	2 × 10^−2^	I
TCEP	4000 ^b^	400	6.84 ^a^	2 × 10^−3^	I	2 × 10^−2^	I	4767	1.19	M	11.9	H	-	-	-
TCPP	30,000 [84]	3000	524	2 × 10^−2^	I	0.2	L	1065	4 × 10^−2^	I	0.36	L	132	4 × 10^−3^	I
TBEP	13,000 [84]	1300	25.6	2 × 10^−3^	I	2 × 10^−2^	I	79.2	6 × 10^−3^	I	6 × 10^−2^	I	20.8	2 × 10^−3^	I
ΣRQ_site_				361					455					723	

mw: marine water (PNEC_fw_/10) from NORMAN Ecotoxicology Database. ^a^ Half of the method LOQ; ^b^ PNEC from NORMAN Ecotoxicology Database. -: not detected.

## Data Availability

The data presented in this study are available in this article and the Supplementary Materials.

## Supplementary Materials

The following supporting information can be downloaded at: https://www.mdpi.com/article/10.3390/molecules28165994/s1, Section S1. Materials and methods: Table S1. Classification of endocrine-disrupting (EDCs) and related compounds analysed in this study with their chemical structure (ChemDraw 21.0.0) and respective physicochemical properties; Figure S1. Central-catchment sampling collection point (red pointer) in the R. Liffey (Dublin, Ireland) and, downstream, Ringsend WWTP (blue pointer); Figure S2. Central-catchment sampling collection point (red pointer) in the R. Thames (London, UK) and six WWTPs discharging straight into the tidal river (blue pointers). Insert shows sewer storm overflow vents (green pointers) in closets in proximity to the sampling collection location (red pointer); Figure S3. DO, conductivity (a), ammonium ion concentration, temperature, and pH (b) monitoring data of the R. Thames on the Putney site during the sample campaign. Data were taken at 15-min intervals; Figure S4. Sampling collection point (red pointer) in the R. Ter (Girona, Spain) and Girona WWTP, on the left, upstream location and another WWTP downstream the sampling point (blue pointers); Table S2. River flow data for the R. Ter extracted from the Agència Catalana de l’Aigua (Catalan Water Agency) online platform for the sampling dates at the 170792-002 monitoring station (coordinates: 485021 X—4648705 Y); Table S3. Summary of monitoring dates for the three river locations; Table S4. Summary of MRM transitions for both negative (−) and positive (+) modes; Sample filtration details; Chromatographic method details; Table S5. Recovery data (±%RSD) and limits of detection (LOD) and quantification (LOQ) for the three river matrices investigated in this study (R. Liffey, R. Thames, and R. Ter); Chromatographic method details; Figure S5. Time series graph of daily total rainfall data (mm) from (a) Girona (Girona Airport Station) for the R. Ter, (b) Dublin (Ringsend Station) for the R. Liffey, and (c) London (Heathrow Station) for the R. Thames during the sampling campaign (dates of sampling are shown in blue arrows). Section S2. Results and Discussion: Table S6. Occurrence (average for *n* = 2 replicates in ng·L^−1^) and frequency of detection (%) of EDCs in surface waters for the R. Liffey (Ireland); Table S7. Occurrence (average for *n* = 2 replicates in ng·L^−1^) and frequency of detection (%) of EDCs in surface waters for the R. Thames (UK); Table S8. Occurrence (average for *n* = 2 replicates in ng·L^−1^) and frequency of detection (%) of EDCs in surface waters for the R. Ter (Spain); Figure S6. E1-3S chromatograms of the quantification MRM transitions from samples of the R. Thames at concentrations <LOQ on the following dates: 20/01/2021 (a), 09/12/2020 (b), and 11/11/2020 (c), a standard at a concentration of 25 ppb (d) and a procedural blank (e); Figure S7. Time series plot of concentrations of quantified compounds in all samples of the R. Thames on the left (ng·L^−1^) with daily total precipitation data (mm) (a) and the high correlation obtained for the triclosan compound (R2 = 0.7138) in the R. Thames between concentrations and rainfall; Figure S8. Principal component analysis (PCA) of the relationship between EDCs detected in the R. Liffey (pink), R. Ter (green), and R. Thames (blue) without caffeine, where the percentage explained by the axes is presented in brackets and concentrations were normalized by compound. References [46,133,134,135,136,137,138,139,140] are cited in the Supplementary Materials.

## Sample Availability

Samples of the compounds are not available from the authors.
