# Supplementary material for: International Comparison, Risk Assessment, and Prioritisation of 26 Endocrine Disrupting Compounds in Three European River Catchments in the UK, Ireland, and Spain
Source: Molecules. 2023 Aug 10;28(16):5994. doi: 10.3390/molecules28165994 (PMC10458904; doi:10.3390/molecules28165994)
Supplement: Supplementary file 1 [file molecules-28-05994-s001.zip › molecules-2502752-supplementary.pdf]

Supplementary Materials:

International Comparison, Risk Assessment, and Prioritisation of 26 Endocrine Disrupting Compounds in Three European River Catchments in the UK, Ireland, and Spain

Helena Rapp-Wright [1,2,3,*], Sara Rodríguez-Mozaz [4,5], Diana Álvarez-Muñoz [4,5], Damià Barceló [4,5,6], Fiona Regan [1,2], Leon P. Barron [3,*] and Blánaid White [1,2]

[1] DCU Water Institute, Water Hub SG57, Dublin City University, Glasnevin, Dublin 9, Dublin, Ireland
[2] School of Chemical Sciences, Dublin City University, Glasnevin, Dublin 9, Ireland
[3] MRC Centre for Environment and Health, Environmental Research Group, School of Public Health, Imperial College London, Wood Lane, London W12 0BZ, United Kingdom
[4] Catalan Institute for Water Research (ICRA-CERCA), C/Emili Grahit 101, 17003 Girona, Spain
[5] University of Girona (UdG), 17004, Girona, Spain
[6] Institute of Environmental Assessment and Water Research (IDAEA-CSIC), 08034 Barcelona, Spain

* Correspondence: H.R.W. h.rapp-wright@imperial.ac.uk and L.P.B. leon.barron@imperial.ac.uk

Section S1. Materials and methods

Table S1. Classification of endocrine-disrupting (EDCs) and related compounds analysed in this study with their chemical structure (ChemDraw 21.0.0) and respective physicochemical properties.

**EDC compound class and name** *(CAS number)*	**Chemical structure**	**LogP**	**LogS (pH = 7.00)**	**No. of H Bond Donors**	**No. of H Bond Acceptors**	**Mw**	**C Ratio**	**NO Ratio**	**Halogen Ratio**	**No. of Rings**	**Log (Koc)**	**pKa(Acid)**			**pKa(Base)**		
												**pKa**	**Conf. limits**	**Atom No**	**pKa**	**Conf. limits**	**Atom No**
**Steroids**<br>(Natural and synthetic human estrogens and conjugates)																	
17-β-estradiol (E2) *(50-28-2)*	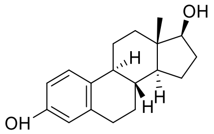	3.6	-4.56	2	2	272.3	0.9	0.1	0	4	3.62	10.27<br>15.09	0.6<br>0.4	20<br>11			
Estriol (E3) *(50-27-1)*	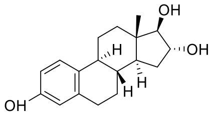	2.7	-4.36	3	3	288.4	0.86	0.14	0	4	2.98	10.25<br>14.55<br>15.55	0.7<br>0.6<br>0.6	21<br>11<br>12			
17-α-ethinylestradiol (EE2) *(57-63-6)*	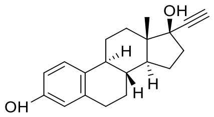	3.9	-4.7	2	2	296.4	0.91	0.09	0	4	3.84	10.24<br>13.12	0.6<br>0.4	22<br>13			
Estriol 3-sufate (E3-3S) *(481-95-8)*	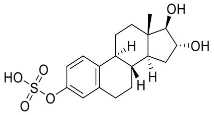	0.9	-1.35	3	6	368.5	0.72	0.24	0	4	2.88	-3.82<br>14.53<br>15.53	0.18<br>0.6<br>0.6	25<br>11<br>12			
Estrone 3-sulfate (E1-3S) *(438-67-5)*	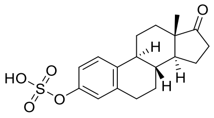	1.6	-1.45	1	5	350.4	0.75	0.21	0	4	3.29	-3.84	0.18	24			

Estrone (E1)<br>*(53-16-7)*	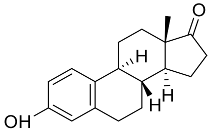	3.4	-4.34	1	2	270.4	0.9	0.1	0	4	3.38	10.25	0.4	20
Testosterone<br>*(58-22-0)*	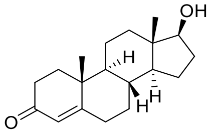	3.2	-4.14	1	2	288.4	0.9	0.1	0	4	3.27	15.06	0.6	11
Progesterone<br>*(57-83-0)*	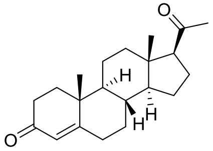	3.7	-4.51	0	2	314.5	0.91	0.09	0	4	3.57			
**Preservatives**														
Methylparaben<br>(MeP)<br>*(99-76-3)*	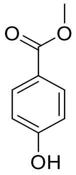	2.1	-1.96	1	3	152.2	0.73	0.27	0	1	2.39	8.31	0.13	11
Ethyllparaben<br>(EtP)<br>*(120-47-8)*	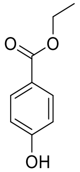	2.5	-2.25	1	3	166.2	0.75	0.25	0	1	2.68	8.31	0.13	12
Propylparaben<br>(PrP)<br>*(94-13-3)*	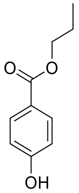	2.9	-2.48	1	3	180.2	0.77	0.23	0	1	2.97	8.23	0.15	13

Benzylparaben<br>(BeP)<br>*(94-18-8)*	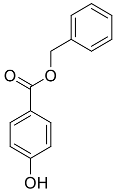 	3.5	-3.53	1	3	228.2	0.82	0.18	0	2	3.36	8.18	0.15	17
**Plasticizers**<br>(Industrial production of polycarbonates and epoxy resins)														
Bisphenol A<br>(BPA)<br>*(80-05-7)*	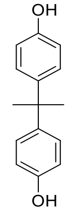 	3.6	-3.24	2	2	228.3	0.88	0.12	0	2	3.24	10.29<br>10.93	0.1<br>0.1	17<br>10
Bisphenol B<br>(BPB)<br>*(77-40-7)*	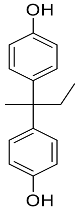 	3.9	-3.39	2	2	242.3	0.89	0.11	0	2	3.53	10.27<br>10.91	0.1<br>0.1	18<br>11
Bisphenol F<br>(BPF)<br>*(620-92-8)*	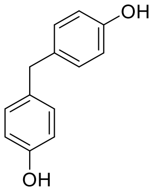 	2.9	-2.71	2	2	200.2	0.87	0.13	0	2	2.86	9.91<br>10.54	0.1<br>0.1	15<br>14
Bisphenol S<br>(BPS)<br>*(80-09-1)*	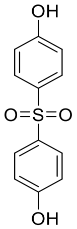 	1.8	-2.29	2	4	250.3	0.71	0.24	0	2	2.37	8.23<br>7.64	0.26<br>0.15	17<br>7

Bisphenol AF<br>(BPAF)<br>*(1478-61-1)*	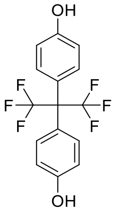	3.4	-5.12	2	2	336.2	0.65	0.09	0.26	2	2.91	8.74<br>9.38	0.1<br>0.1	23<br>14
**Alkylphenols**<br>(Manufacture of household and industrial products)														
Nonylphenol<br>(NP)<br>*(104-40-5)*	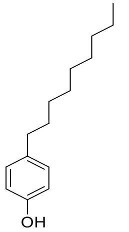	6.1	-5.11	1	1	220.4	0.94	0.06	0	1	4.75	10.15	0.15	16
Octylphenol<br>(OP)<br>*(1806-26-4)*	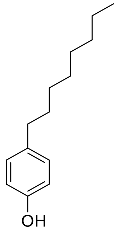	5.5	-4.53	1	1	206.3	0.93	0.07	0	1	4.46	10.15	0.15	15
**Organo-phosphorus and brominated-based flame retardants**														
Tris(butoxyethy<br>l) phosphate<br>(TBEP)<br>*(78-51-3)*	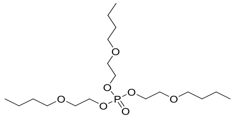	3.5	-2.63	0	7	398.5	0.69	0.27	0	0	3.71			
Tris(2-<br>chloroisopropyl<br>) phosphate<br>(TCPP)<br>*(13674-84-5)*	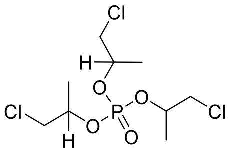	2.3	-2.43	0	4	327.6	0.53	0.24	0.18	0	2.21			

Tris(2-chloroethyl) phosphate (TCEP) *(115-96-8)*	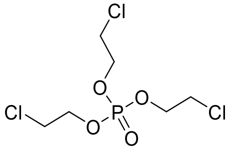	1.4	-1.75	0	4	285.5	0.43	0.29	0.21	0	1.64						
Tetrabromobis phenol A (TBBPA) *(79-94-7)*	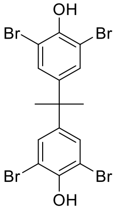	6.8	-7.01	2	2	543.9	0.71	0.1	0.19	2	5.34	8.5<br>9.14	0.1<br>0.1	20<br>11			
**Other compounds**																	
Triclosan *(3380-34-5)* *(Antimicrobial/desinfectant)*	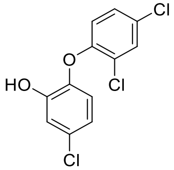	5.3	-4.87	1	2	289.5	0.71	0.12	0.18	2	4.19	7.8	0.35	8			
1H-Benzotriazole (BT) *(95-14-7)* *(Anticorrosive)*	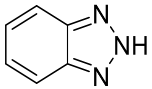	1.5	-1.44	1	3	119.1	0.67	0.33	0	2	2	8.38	0.1	5	1.47	0.3	6
Caffeine *(58-08-2)*	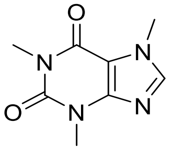	0.3	-0.83	0	6	194.2	0.57	0.43	0	2	1.31				0.52<br>-3.83	0.7<br>0.2	4<br>9

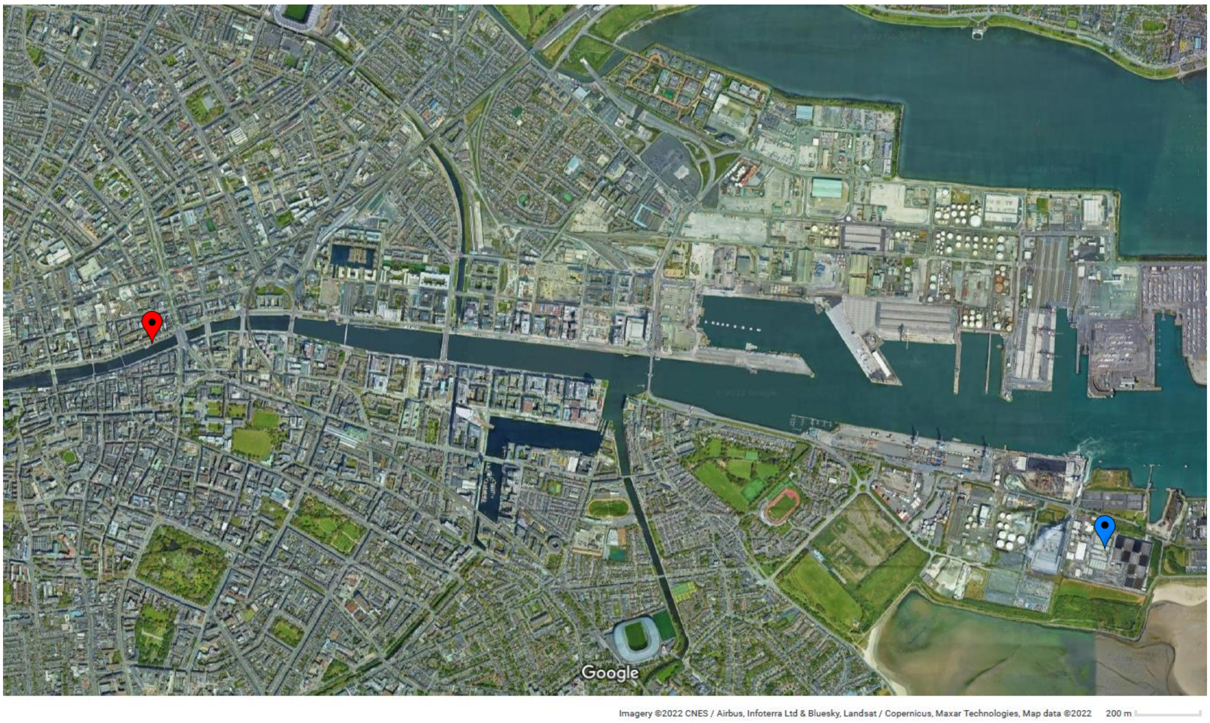

Figure S1. Central-catchment sampling collection point (red pointer) in the R. Liffey (Dublin, Ireland) and, downstream, Ringsend WWTP (blue pointer).

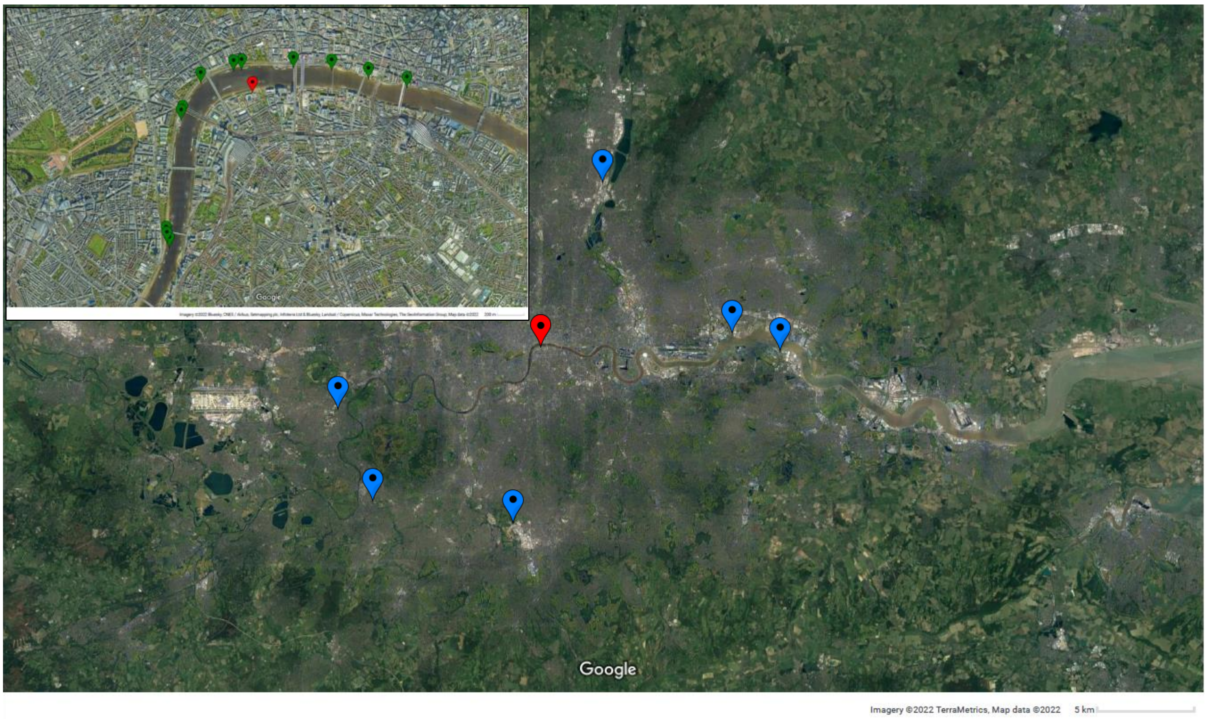

Figure S2. Central-catchment sampling collection point (red pointer) in the R. Thames (London, UK) and six WWTPs discharging straight into the tidal river (blue pointers). Insert shows sewer storm overflow vents (green pointers) in closets proximity to the sampling collection location (red pointer).

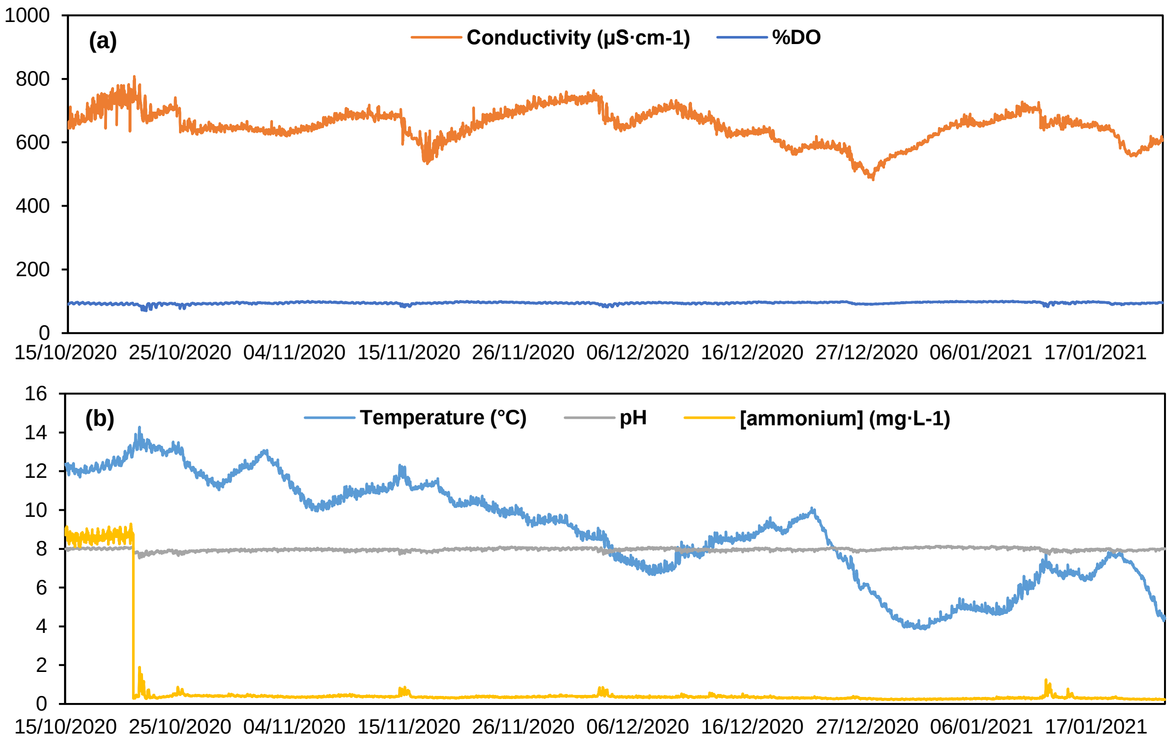

Figure S3. DO, conductivity (a), ammonium ion concentration, temperature, and pH (b) monitoring data of the R. Thames on the Putney site during the sample campaign. Data taken every 15-minute intervals.

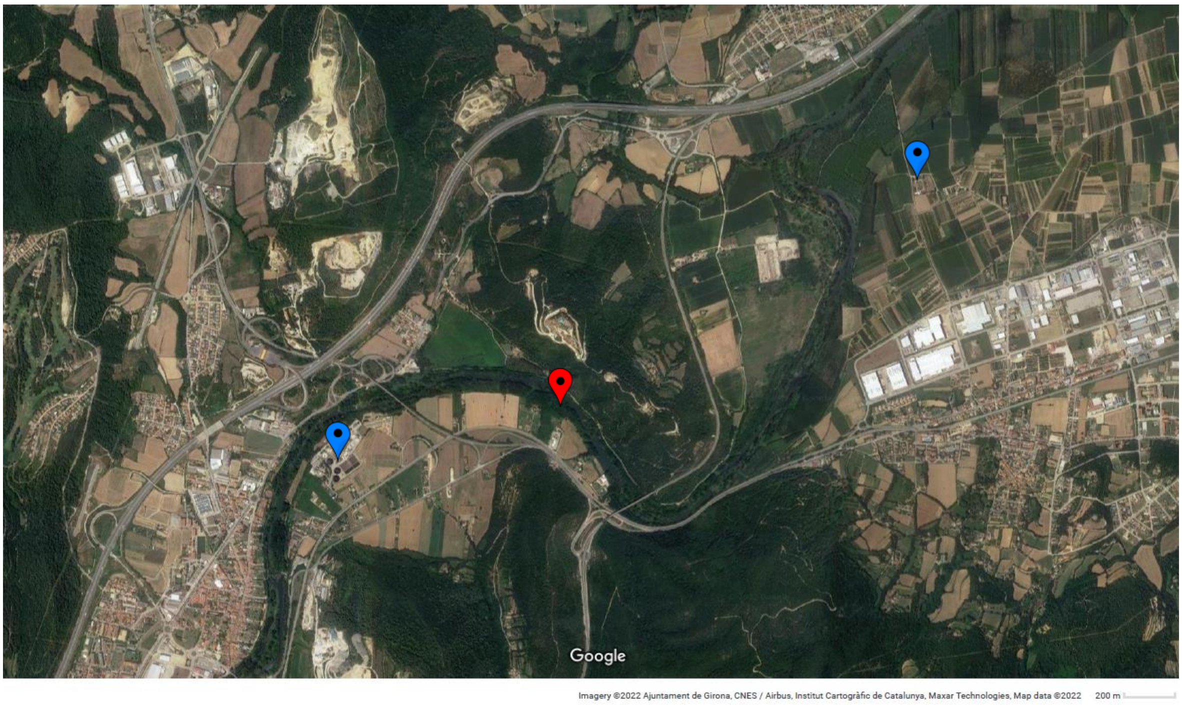

Figure S4. Sampling collection point (red pointer) in the R. Ter (Girona, Spain) and Girona WWTP, on the left, upstream location and another WWTP downstream the sampling point (blue pointers).

Table S2. River flow data for the R. Ter extracted from the Agència Catalana de l'Aigua (Catalan Water Agency) online platform for the sampling dates at the 170792-002 monitoring station (coordinates: 485021 X – 4648705 Y).

Sampling date	Flow (m^3/day)
23/10/20	342,097
28/10/20	332,640
02/11/20	458,033
11/11/20	579,664
18/11/20	372,604
25/11/20	439,496
02/12/20	3691,374
08/12/20	535,177
17/12/20	336,096
Mean	787,465
Median	439,496

Table S3. Summary of monitoring dates for the three river locations.

	Sampling dates									
R. Liffey	23/10/20	28/10/20	02/11/20	11/11/20	18/11/20	25/11/20	02/12/20	08/12/20	17/12/20	20/01/21
R. Ter	21/10/20	28/10/20	04/11/20	11/11/20	18/11/20	25/11/20	02/12/20	09/12/20	16/12/20	20/01/21
R. Thames	23/10/20	28/10/20	02/11/20	11/11/20	18/11/20	25/11/20	02/12/20	09/12/20	16/12/20	20/01/21

Table S4. Summary of MRM transitions for both negative (-) and positive (+) modes.

Compounds	Precursor ion (m/z)	Product ions (m/z)		t_R (min)	Polarity	SIL-IS
Caffeine	195	138	Quan	3.76	+	Caffeine-d_3
		110	Qual		+	-
Caffeine-d_3	198	138	Quan	3.75	+	-
		110	Qual		+	
BT	120	65	Quan	3.91	+	BT-d_4
		92	Qual		+	-
BT-d_4	124	69	Quan	3.89	+	-
		96	Qual		+	-
TCEP	287	98	Quan	4.34	+	Triphenylphosphate-d_{15}
		225	Qual		+	-
TCPP	327	98	Quan	4.71	+	Triphenylphosphate-d_{15}
		80	Qual		+	-
TBEP	399	299	Quan	5.34	+	Triphenylphosphate-d_{15}
		199	Qual		+	-
Triphenylphosphate-d_{15}	342	160	Quan	4.97	+	-
		159	Qual		+	-
Testosterone	289	97	Quan	4.82	+	Progesterone-d_9
		109	Qual		+	-
Progesterone	315	97	Quan	5.10	+	Progesterone-d_9
		109	Qual		+	-
Progesterone-d_9	323	100	Quan	5.10	+	-
Caffeine $C^{13}{}_3$ (surrogate)	198	140	Quan	3.80	+	-
		112	Qual		+	-
E1	269	145	Quan	6.37	-	E1-d_4
		143	Qual		-	-
E1-d_4	273	145	Quan	6.37	-	-
		147	Qual		-	-
E2	271	183	Quan		-	E2-d_2
		145	Qual	6.39	-	-
E2-d_2	273	147	Quan	6.36	-	-

E3	287	171	Quan	5.18	-	E2-d_2
		145	Qual		-	-
EE2	295	145	Quan	6.36	-	EE2-d_4
		159	Qual		-	-
EE2-d_4	299	147	Quan	6.35	-	-
		161	Qual		-	-
E3-S1	367	287	Quan	3.61	-	E2-d_2
		171	Qual		-	-
E3-S3	349	269	Quan	4.69	-	E1-d_4
		145	Qual		-	-
MeP	151	92	Quan	4.63	-	MeP-d_4
		136	Qual		-	-
MeP-d_4	154	96	Quan	4.60	-	-
EtP	165	92	Quan	5.29	-	MeP-d_4
		137	Qual		-	-
PrP	179	92	Quan	5.86	-	MeP-d_4
		136	Qual		-	-
BeP	227	92	Quan	6.32	-	MeP-d_4
		136	Qual		-	-
OP	205	106	Quan	7.79	-	OP-d_{17}
OP-d_{17}	222	108	Quan	7.76	-	-
NP	219	106	Quan	8.01	-	NP-d_4
NP-d_4	223	110	Quan	8.00	-	-
Triclosan	286	35	Quan	7.33	-	Triclosan-d_3
Triclosan-d_3	289	35	Quan	7.33	-	-
BPA	227	212	Quan	5.92	-	BPA-d_4
		133	Qual		-	-
BPA-d_4	231	216	Quan	5.91	-	-
BPB	241	212	Quan	6.27	-	BPB-d_8
		211	Qual		-	-
BPB-d_8	249	219	Quan	6.24	-	-
		220	Qual		-	-
BPF	199	93	Quan	5.33	-	BPS-d_8
		105	Qual		-	-
BPS	249	108	Quan	4.33	-	BPS-d_8
		92	Qual		-	-
BPS-d_8	257	96	Quan	4.29	-	-
		112	Qual		-	-
BPAF	335	265	Quan	6.51	-	BPS-d_8
		197	Qual		-	-
TBBPA	542	418	Quan	7.38	-	BPA-d_4
		445	Qual		-	-
EtP $^{13}C_6$ (surrogate)	170	98	Quan	5.26	-	-
		143	Qual		-	-

Quan: quantification ion.
Qual: qualifier ion.

Sample filtration details:

In order to remove particles from aqueous samples, the use of microporous membrane filters is a simple and effective method. In this study, river water samples had high suspended solids and therefore, samples were filtered twice using two different pore sizes. First, a higher pore size (0.7 μm) was used and then a smaller one (0.45 μm), this allows the sample to go through without blocking the filter by using the higher pore size first. The 0.45 μm pore size is needed to remove smaller particles that can block the solid-phase extraction (SPE) cartridges used for extraction. Moreover, glass fibre filters are commonly used as the primary filter in environmental water samples for analysis of contaminants of emerging concern due to a large variety of pore sizes available and their low cost (Hidayati et al., 2021; Jardim et al., 2012); also removing trace metals (Fuhrmann and Fitts, 2004) that can cause interferes with some of the analytes selected when analysed. PVDF filters are also regularly used for water samples (Alonso et al., 2022; Alsohaimi et al., 2012; Santana-Mayor et al., 2021) targeting compounds such as pharmaceuticals (Boltner et al., 2016; Carlson and Thompson, 2000) due to their high solid particle retention and by lowering the levels of extractable impurities.

Chromatographic method details:

Chromatographic separations were achieved using mobile phases of HPLC grade methanol (A) and water (B) with two different gradients for the negative and positive ionisation modes. The negative mode was utilized applying 20 µL sample injection volume, where mobile phases were at a constant flow rate of 0.4 mL/min for a total run time of 10.5 minutes. At starting conditions, A was set at 20 % for a minute; 1-2.75 min: another linear ramp of A increased to 50 %; 2.75-6.50 min: A further increased to 100 %; 6.50-8: A stayed at 100 %; 8-9.50: A returned to the initial conditions of 20 % and maintained there for 1 extra minute. On the other hand, the positive mode method was performed using an injection volume of 10 µL applying the same mobile phases at a constant flow rate set at 0.3 mL/min. The gradient elution was as follows: A was set at 10 % for a minute; from 1-2.75 min: A increased to 100 %; 2.75-5.50: A stayed at 100 %; 5.50-6.50: A returned to the initial conditions of 10 % and re-equilibrated for 1 extra minute, resulting in a total run time of 7.5 minutes.

Table S5. Recovery data (±%RSD) and limits of detection (LOD) and quantification (LOQ) for the three river matrices investigated in this study (R. Liffey, R. Thames and R. Ter).

Analtes	Recovery ± %RSD			LOD (ng·L^{-1})			LOQ (ng·L^{-1})		
	**Liffey**	**Thames**	**Ter**	**Liffey**	**Thames**	**Ter**	**Liffey**	**Thames**	**Ter**
Caffeine	115 ± 6	150 ± 5	103 ± 3	7.3	9.8	5.9	24.2	32.8	19.7
BT	101 ± 4	138 ± 8	117 ± 5	1.7	1.9	1.2	5.8	6.2	4.0
TCEP	26 ± 8	17 ± 19	19 ± 15	4.1	6.5	1.8	13.7	21.7	6.1
TCPP	79 ± 12	149 ± 14	64 ± 19	17.3	15.3	9.7	57.6	51.0	32.3
Testosterone	39 ± 12	48 ± 12	40 ± 9	0.1	0.1	0.3	0.4	0.4	0.9
Progesterone	57 ± 14	53 ± 21	68 ± 1	0.1	0.1	0.2	0.4	0.4	0.6
TBEP	78 ± 8	124 ± 19	111 ± 7	1.4	0.3	0.3	4.8	1.0	0.8
E1-3S	95,1*	95,1*	149 ± 0	1.2	2.2	1.0	3.9	7.2	3.3
BPS	146 ± 3	107 ± 5	138 ± 1	0.1	0.04	0.02	0.2	0.1	0.1
E3-S3	n.r.	n.r.	124 ± 0	6.3	14.3	5.0	20.3	47.5	16.6
MeP	90 ± 5	97 ± 7	62 ± 3	5.0	5.9	3.8	16.8	19.8	12.7
E3	74 ± 4	113 ± 10	63 ± 5	8.4	9.8	5.4	27.9	32.7	17.9
EtP	94 ± 3	96 ± 12	69 ± 4	0.6	0.8	0.5	2.1	2.6	1.8
BPF	93 ± 4	92 ± 7	78 ± 0	13.7	18.2	11.0	45.6	60.8	36.7
PrP	91 ± 3	92 ± 13	61 ± 2	1.3	1.2	1.1	4.4	4.0	3.7
BPA	71 ± 4	75 ± 15	48 ± 1	10.8	9.5	5.0	36.0	31.8	16.8
BPB	106 ± 7	88 ± 7	85 ± 3	2.8	1.6	3.7	9.5	5.3	12.3
BeP	92 ± 4	80 ± 20	61 ± 3	1.2	1.2	1.1	4.1	3.9	3.6
EE2	98 ± 13	104,0*	78 ± 19	11.2	6,5*	11.6	37.2	21.8*	38.6
E1	78 ± 13	78 ± 15	72 ± 2	1.3	3.4	0.7	4.9	11.2	2.4
E2	124 ± 8	103 ± 21	79 ± 4	5.3	6.3	7.4	17.7	20.9	24.6
Triclosan	75 ± 11	56 ± 0	48 ± 40	21.1	9.9	2.6	70.2	33.0	8.8
TBBPA	125*	125*	125*	0,1*	0,1*	0,1*	0,3*	0,3*	0,3*
OP	59*	59*	59*	6,0*	6,0*	6,0*	20*	20*	20*
NP	24*	24*	24*	7.4*	7.4*	7.4*	25*	25*	25*
BPAF	108 ± 13	57 ± 20	67 ± 2	0.2	0.3	0.1	0.9	0.8	0.5

*from method development (Becker et al., 2017).
n.r. = not recovered.

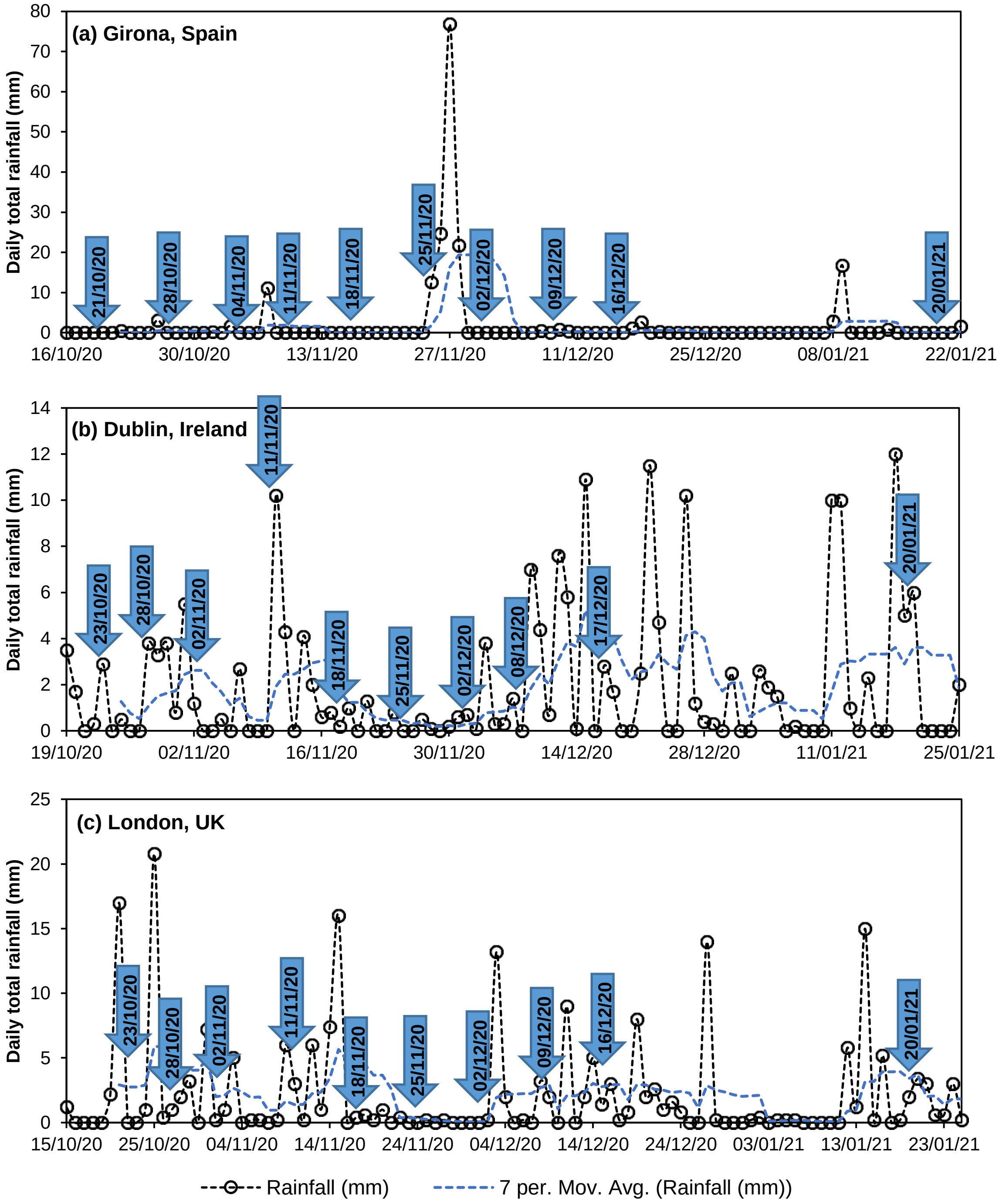

Figure S5. Time series graph of daily total rainfall data (mm) from (a) Girona (Girona Airport Station) for the R. Ter, (b) Dublin (Ringsend Station) for the R. Liffey and (c) London (Heathrow Station) for the R. Thames during the sampling campaign (dates of sampling are shown in blue arrows).

Section S2. Results and Discussion

Table S6. Occurrence (average for n=2 replicates in ng·L^{-1}) and frequency of detection (%) of EDCs in surface waters for the R. Liffey (Ireland).

Analyte	River Liffey										Frequency (%)
	23/10/20	28/10/20	02/11/20	11/11/20	18/11/20	25/11/20	02/12/20	08/12/20	17/12/20	20/01/21	
Testosterone	3.63	-	-	-	-	-	-	-	-	<LOQ	20
Progesterone	-	0.6	-	-	-	-	-	-	-	-	10
E1	-	-	<LOQ	-	-	<LOQ	-	-	-	<LOQ	30
E2	-	-	-	-	-	-	-	-	-	-	0
E1-3S	-	-	-	-	<LOQ	<LOQ	-	-	-	-	20
E3-3S	-	-	-	-	-	-	-	-	-	-	0
E3	-	-	-	-	-	-	-	-	-	-	0
EE2	-	-	-	-	-	-	-	-	-	-	0
Caffeine	73.7	338	192	128	139	102	58.2	57.8	65.9	151	100
Triclosan	<LOQ	-	<LOQ	<LOQ	<LOQ	-	-	-	<LOQ	<LOQ	60
MeP	<LOQ	-	-	-	-	-	-	-	-	-	10
EtP	3.69	<LOQ	2.91	2.34	<LOQ	<LOQ	<LOQ	3.34	<LOQ	2.53	100
PrP	-	-	-	-	-	-	-	-	-	-	0
BeP	-	-	-	-	-	-	-	-	-	-	0
BPA	<LOQ	-	-	-	-	-	-	<LOQ	-	-	20
BPB	-	-	-	-	-	-	-	-	-	-	0
BPF	-	-	-	-	-	-	-	-	-	-	0
BPS	-	-	-	-	-	-	-	13.0	-	5.40	20
BPAF	-	-	-	-	-	-	-	-	-	-	0
OP	-	-	-	-	-	-	-	-	-	-	0
NP	-	-	-	-	-	-	-	-	-	-	0
BT	75.0	74.1	102	127	90.5	131	156	218	100	125	100
TCEP	<LOQ	<LOQ	-	-	-	-	-	-	<LOQ	<LOQ	40
TCPP	107	187	524	150	161	277	221	293	222	322	100
TBEP	11.2	7.55	14.0	7.39	13.1	25.6	5.87	5.13	8.57	6.19	100
TBBPA	-	-	-	-	-	-	-	-	-	-	0

-: not detected.

<LOQ: concentration obtained lower than the limit of quantification.

Table S7. Occurrence (average for n=2 replicates in ng·L^{-1}) and frequency of detection (%) of EDCs in surface waters for the R. Thames (UK).

**Analyte**	**River Thames**										**Frequency**
	**23/10/20**	**28/10/20**	**02/11/20**	**11/11/20**	**18/11/20**	**25/11/20**	**02/12/20**	**09/12/20**	**16/12/20**	**20/01/21**	**(%)**
Testosterone	-	-	-	-	-	<LOQ	-	-	-	-	10
Progesterone	-	-	-	-	-	-	-	-	-	-	0
E1	-	-	-	-	-	<LOQ	-	-	-	<LOQ	20
E2	-	-	-	-	-	-	-	-	-	-	0
E1-3S	-	<LOQ	<LOQ	<LOQ	<LOQ	<LOQ	-	<LOQ	<LOQ	<LOQ	80
E3-3S	-	-	-	-	-	-	-	-	-	-	0
E3	-	-	-	-	-	-	-	-	-	-	0
EE2	-	-	-	-	-	-	-	-	-	-	0
Caffeine	331	324	259	432	274	132	130	344	246	300	100
Triclosan	35.7	40.6	37.7	36.2	-	-	-	-	75.8	-	50
MeP	-	<LOQ	-	-	-	<LOQ	<LOQ	<LOQ	-	-	40
EtP	<LOQ	<LOQ	<LOQ	<LOQ	-	<LOQ	19.5	12.8	7.82	<LOQ	90
PrP	-	-	-	-	-	-	-	-	-	-	0
BeP	-	-	-	-	-	-	-	-	-	-	0
BPA	<LOQ	-	-	-	<LOQ	<LOQ	-	-	-	<LOQ	40
BPB	-	-	-	-	-	-	-	-	-	-	0
BPF	-	-	-	-	-	-	-	-	-	-	0
BPS	60.0	19.2	26.7	14.0	2.87	16.4	7.73	1.87	20.8	79.3	100
BPAF	-	-	-	-	-	-	-	-	-	-	0
OP	-	-	-	-	-	-	-	-	-	-	0
NP	-	-	-	-	-	-	-	-	-	<LOQ	10
BT	357	301	276	186	193	173	351	340	353	258	100
TCEP	62.3	4767	268	274	1034	1033	<LOQ	<LOQ	<LOQ	116	100
TCPP	431	1065	402	445	523	692	257	469	298	559	100
TBEP	65.8	75.6	25.4	79.2	23.3	46.0	22.4	66.9	32.9	41.1	100
TBBPA	-	-	-	-	-	-	-	-	-	-	0

-: not detected.

<LOQ: concentration obtained lower than the limit of quantification.

Table S8. Occurrence (average for n=2 replicates in ng·L^{-1}) and frequency of detection (%) of EDCs in surface waters for the R. Ter (Spain).

**Analyte**	**River Ter**										**Frequency (%)**
	**21/10/20**	**28/10/20**	**04/11/20**	**11/11/20**	**18/11/20**	**25/11/20**	**02/12/20**	**09/12/20**	**16/12/20**	**20/01/21**	
Testosterone	-	-	-	<LOQ	<LOQ	-	-	<LOQ	<LOQ	-	40
Progesterone	-	-	-	-	-	<LOQ	5.4	-	-	-	20
E1	-	-	30.6	-	-	-	-	15.2	30.0	-	30
E2	-	-	-	-	-	-	-	-	-	-	0
E1-3S	-	-	-	-	-	-	-	-	-	-	0
E3-3S	-	-	-	-	-	-	-	-	-	-	0
E3	-	-	-	-	-	-	-	-	-	-	0
EE2	-	-	-	-	-	-	-	-	-	-	0
Caffeine	111	705	107	429	112	105	33.6	184	199	148	100
Triclosan	-	<LOQ	-	-	<LOQ	<LOQ	<LOQ	-	<LOQ	-	50
MeP	-	-	-	39.2	<LOQ	<LOQ	<LOQ	-	-	-	40
EtP	<LOQ	<LOQ	<LOQ	9.53	7.86	8.92	<LOQ	<LOQ	-	-	80
PrP	-	-	-	-	-	-	-	-	-	-	0
BeP	-	-	-	-	-	-	-	-	-	-	0
BPA	-	-	-	-	<LOQ	<LOQ	-	<LOQ	-	<LOQ	40
BPB	-	-	-	-	-	-	-	-	-	-	0
BPF	-	-	-	-	-	-	-	-	-	<LOQ	10
BPS	-	2.61	9.31	4.68	8.24	3.62	-	0.24	2.10	79.3	80
BPAF	-	-	-	-	-	-	36.9	-	-	-	10
OP	-	-	-	-	26.9	-	54.1	54.0	26.8	-	40
NP	-	-	-	-	-	-	-	-	-	-	0
BT	129	94.7	93.5	102	86.7	94.2	50.3	62.7	117	136	100
TCEP	-	-	-	-	-	-	-	-	-	-	0
TCPP	63.9	132	71.2	117	111	<LOQ	<LOQ	<LOQ	<LOQ	47.6	100
TBEP	14.7	7.59	6.30	20.8	18.4	<LOQ	16.5	<LOQ	<LOQ	8.36	100
TBBPA	-	-	-	-	-	-	-	-	-	-	0

-: not detected.

<LOQ: concentration obtained lower than the limit of quantification.

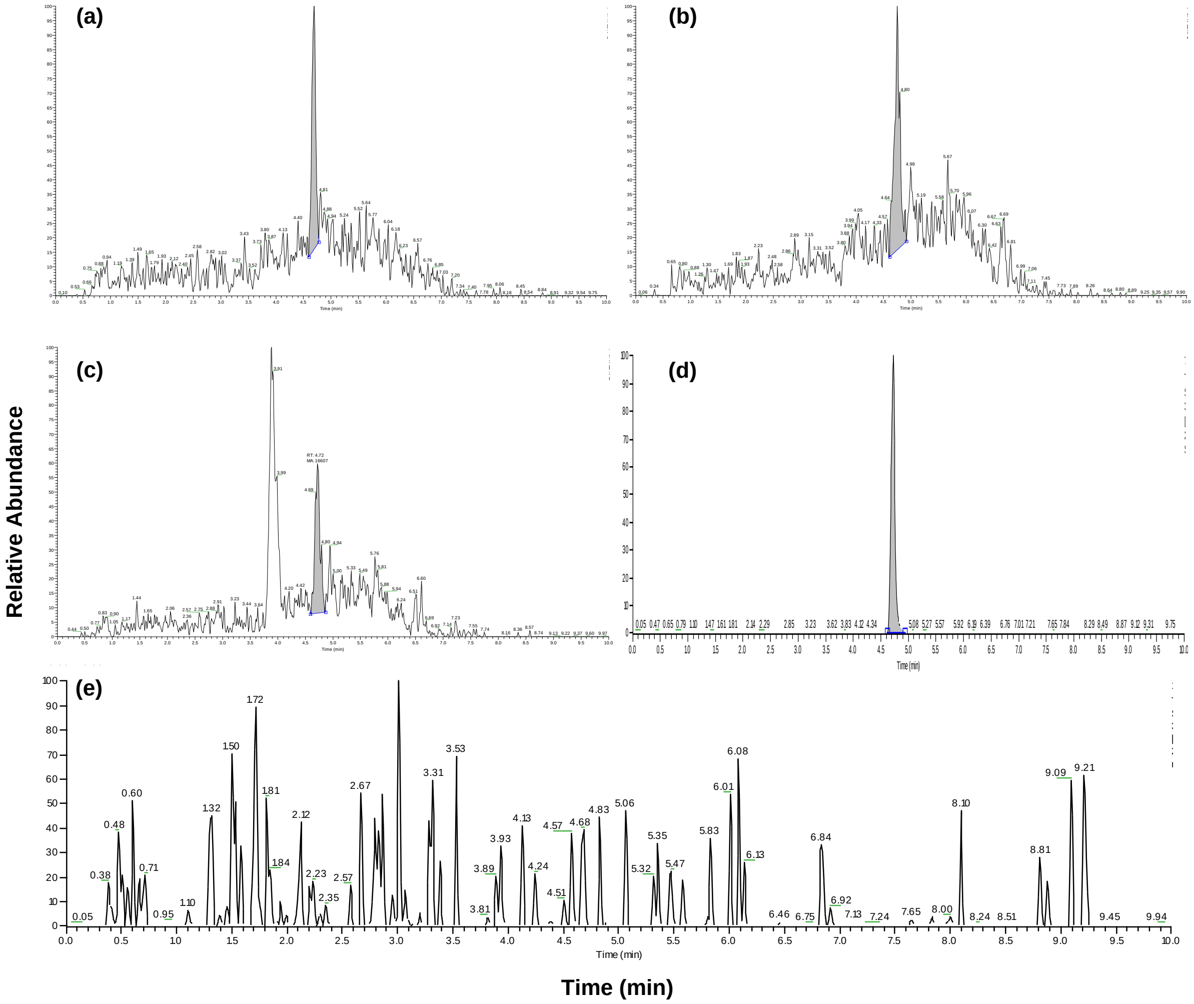

Figure S6. E1-3S chromatograms of the quantification MRM transitions from samples of the R. Thames at concentrations <LOQ on the following dates: 20/01/2021 (a), 09/12/2020 (b) and 11/11/2020 (c), a standard at a concentration of 25 ppb (d) and a procedural blank (e).

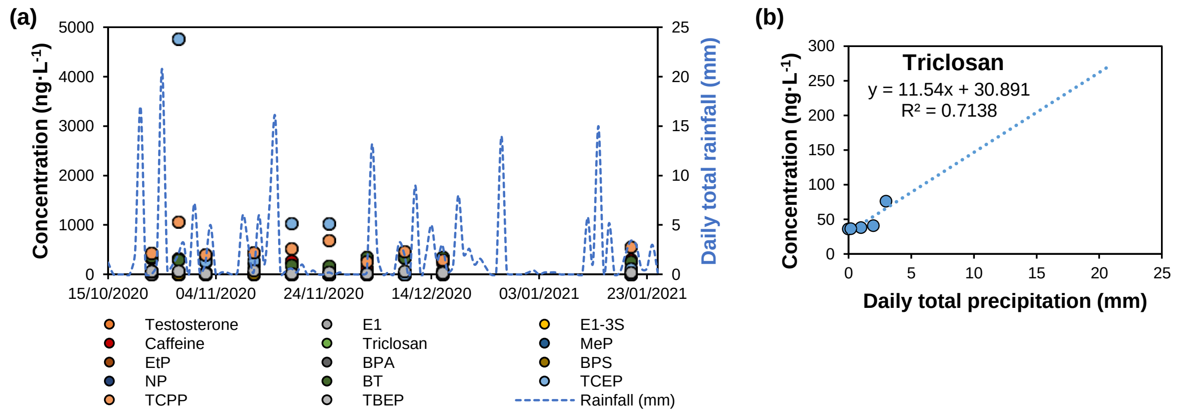

Figure S7. Time series plot of concentrations of quantified compounds in all samples of the R. Thames on the left (ng·L^{-1}) with daily total precipitation data (mm) (a) and the high correlation obtained for triclosan compound (R^2=0.7138) in the R. Thames between concentrations and rainfall.

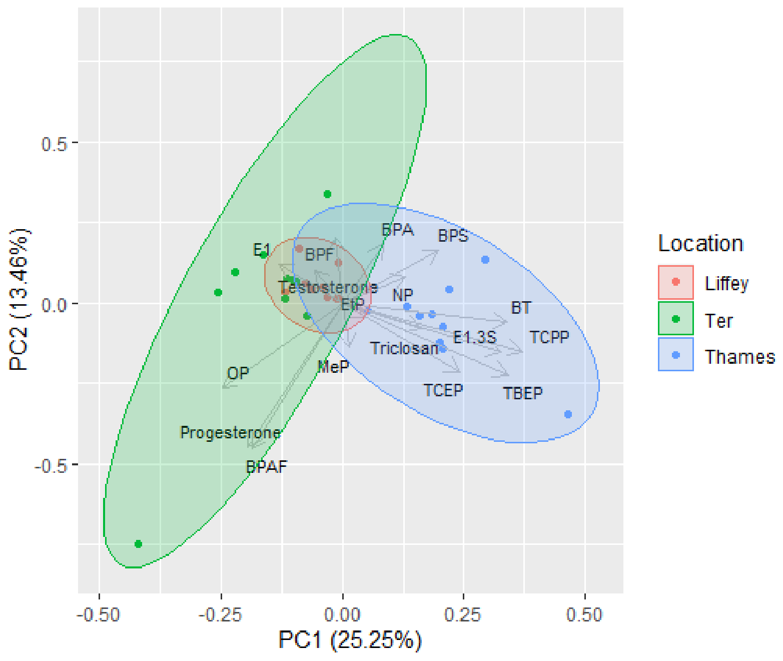

Figure S8. Principal component analysis (PCA) of the relationship between EDCs detected in the R. Liffey (pink), R. Ter (green) and R. Thames (blue) without caffeine; where the percentage explained by the axes is presented in brackets and concentrations were normalised by compound.

References

Alonso, B., Griffero, L., Bentos Pereira, H., Pareja, L., Pérez Parada, A., 2022. Determination of glyphosate and AMPA in freshwater and soil from agroecosystems by 9-fluorenylmethoxycarbonyl chloride derivatization and liquid chromatography - fluorescence detection and tandem mass spectrometry. MethodsX 9, 101730. https://doi.org/10.1016/j.mex.2022.101730

Alsohaimi, I.H., Alothman, Z.A., Khan, M.R., Abdalla, M.A., Busquets, R., Alomary, A.K., 2012. Determination of bromate in drinking water by ultraperformance liquid chromatography-tandem mass spectrometry: Liquid Chromatography. J. Sep. Science 35, 2538–2543. https://doi.org/10.1002/jssc.201200312

Becker, D., Rodriguez-Mozaz, S., Insa, S., Schoevaart, R., Barceló, D., de Cazes, M., Belleville, M.-P., Sanchez-Marcano, J., Misovic, A., Oehlmann, J., Wagner, M., 2017. Removal of Endocrine Disrupting Chemicals in Wastewater by Enzymatic Treatment with Fungal Laccases. Org. Process Res. Dev. 21, 480–491. https://doi.org/10.1021/acs.oprd.6b00361

Boltner, A., Schröder, W., Grosse, S., Schreiber, A., Letzel, T., 2016. Simultaneous Characterization of Highly Polar, Polar and Nonpolar Compounds in River Water using Serial Coupled RPLC and HILIC with a QTRAP® 5500 LC-MS/MS - Identification using MRM Ratios and Enhanced Product Ion Scanning (EPI).

Carlson, M., Thompson, R.D., 2000. Analyte Loss Due to Membrane Filter Adsorption as Determined by High-Performance Liquid Chromatography. Journal of Chromatographic Science 38, 77–83. https://doi.org/10.1093/chromsci/38.2.77

Fuhrmann, M., Fitts, J.P., 2004. Adsorption of Trace Metals on Glass Fiber Filters. J. environ. qual. 33, 1943–1944. https://doi.org/10.2134/jeq2004.1943

Hidayati, N.V., Syakti, A.D., Asia, L., Lebarillier, S., Khabouchi, I., Widowati, I., Sabdono, A., Piram, A., Doumenq, P., 2021. Emerging contaminants detected in aquaculture sites in Java, Indonesia. Science of The Total Environment 773, 145057. https://doi.org/10.1016/j.scitotenv.2021.145057

Jardim, W.F., Montagner, C.C., Pescara, I.C., Umbuzeiro, G.A., Di Dea Bergamasco, A.M., Eldridge, M.L., Sodré, F.F., 2012. An integrated approach to evaluate emerging contaminants in drinking water. Separation and Purification Technology 84, 3–8. https://doi.org/10.1016/j.seppur.2011.06.020

Santana-Mayor, Á., Socas-Rodríguez, B., Rodríguez-Ramos, R., Herrera-Herrera, A.V., Rodríguez-Delgado, M.Á., 2021. Quality assessment of environmental water by a simple and fast non-ionic hydrophobic natural deep eutectic solvent-based extraction procedure combined with liquid chromatography tandem mass spectrometry for the determination of plastic migrants. Anal Bioanal Chem 413, 1967–1981. https://doi.org/10.1007/s00216-021-03166-1
